# Resolving the central metabolism of Arabidopsis guard cells

**DOI:** 10.1038/s41598-017-07132-9

**Published:** 2017-08-16

**Authors:** Semidán Robaina-Estévez, Danilo M. Daloso, Youjun Zhang, Alisdair R. Fernie, Zoran Nikoloski

**Affiliations:** 1 Systems Biology and Mathematical Modeling Group, Max Planck Institute of Molecular Plant Physiology, Potsdam, Golm Germany; 2 Bioinformatics Group, Institute of Biochemistry and Biology, University of Potsdam, Potsdam, Golm Germany; 3 Central Metabolism Group, Max Planck Institute of Molecular Plant Physiology, Potsdam, Golm Germany; 4 Departamento de Bioquímica e Biologia Molecular, Universidade Federal do Ceará, Fortaleza, CE Brazil

## Abstract

Photosynthesis and water use efficiency, key factors affecting plant growth, are directly controlled by microscopic and adjustable pores in the leaf—the stomata. The size of the pores is modulated by the guard cells, which rely on molecular mechanisms to sense and respond to environmental changes. It has been shown that the physiology of mesophyll and guard cells differs substantially. However, the implications of these differences to metabolism at a genome-scale level remain unclear. Here, we used constraint-based modeling to predict the differences in metabolic fluxes between the mesophyll and guard cells of *Arabidopsis thaliana* by exploring the space of fluxes that are most concordant to cell-type-specific transcript profiles. An independent ^13^C-labeling experiment using isolated mesophyll and guard cells was conducted and provided support for our predictions about the role of the Calvin-Benson cycle in sucrose synthesis in guard cells. The combination of *in silico* with *in vivo* analyses indicated that guard cells have higher anaplerotic CO_2_ fixation via phospho*enol*pyruvate carboxylase, which was demonstrated to be an important source of malate. Beyond highlighting the metabolic differences between mesophyll and guard cells, our findings can be used in future integrated modeling of multi-cellular plant systems and their engineering towards improved growth.

## Introduction

The stomata, microscopic and adjustable pores on the leaf surface, directly control two of the most important parameters affecting plant growth: carbon dioxide (CO_2_) uptake from the environment and transpiration^1^. Thus, knowledge of the processes involved in stomatal movement is fundamental to understanding plant growth, and may represent a route to optimizing crop yield under the increasingly challenging environmental conditions^2, 3^. Stomatal movement depends on variations of the volume of two highly specialized, kidney-shaped cells, the guard (G) cells, which juxtapose to form the pore. The variations in the volume of the G cells are the macroscopic result of an intricate network of molecular processes occurring at different hierarchical scales^4^. G cells stand out from the rest of the epithelial tissue—in which they are embedded—not only for their particular shape, but also for the remarkable property of containing photosynthetically active chloroplasts. Rather than contributing to total leaf carbon fixation, it has been suggested that active chloroplasts may be linked to the particular energetic and metabolic requirements for adequate G cell functioning^5^. In contrast, carbon fixation is the primary task of the main photosynthetically active cells, the mesophyll (M) cells. Although G and M cells are physiologically differentiated, the close connection between stomatal aperture and photosynthetic efficiency likely involves a fine coordination between these two cell types^4, 6^.

G cell represents a multisensorial system that responds to endogenous and environmental signals. Therefore, understanding of the complex cellular processes behind stomatal movement requires a systems approach to integrate experimental data with mathematical description of the underlying mechanisms. In practice, however, a complete mathematical description of stomatal movement is challenging due to experimental challenges and the hierarchy at which the key processes take place. Nevertheless, several studies, focusing on the dynamical processes of stomatal aperture, have rendered promising results derived from small-scale kinetic models^7–9^. For instance, the OnGuard modeling framework has been instrumental for explaining the dynamics of stomatal movement^3, 9–11^. It is based on a system of ordinary differential equations modeling the relationships between the influxes and outfluxes of water and different inorganic and organic osmolytes, the membrane potential, and macroscopic variables such as: total guard cell volume, turgor pressure and stomatal aperture. OnGuard also provides a phenomenological description of the metabolic processes involving the main organic osmolytes: sucrose and malate. However, it remains silent with respect to a detailed description of the genome-scale metabolic processes occurring in G cells, since they are out of the scope of the kinetic modeling approach.

Relatively little is known about the genome-scale metabolic differences between G and M cells, although they can provide key insights into the modulation of metabolism in the two cell types^11^. A genome-scale description of the metabolic state of G cells would provide a valuable complement to the existing kinetic models^7, 12^. To this end, one can use the advances in genome-scale, constraint-based modeling of plants^13–17^, which have facilitated testing of hypotheses concerning the (re)distribution of steady-state metabolic activity under various conditions^18^. Integration of cell-type-specific data in this modeling approach is important since direct measurements of metabolic activity at a systems level are currently infeasible^19, 20^. Transcriptomics data have been successfully employed to derive activity patterns in context-specific metabolic networks across a variety of organisms, from prokaryotes to more complex eukaryotes^21–23^, and are readily available for G and M cells^24–30^.

Constraint-based modeling with integration of transcriptomics data provides a way to conduct differential analysis of metabolic activity between G and M cells. However, using transcriptomic—or even proteomic—data as an indicator of metabolic state calls for further justification, since metabolism is downstream in the cellular hierarchical organization. Integration of transcriptomics data is generally justified by two arguments: (*i*) transcript levels are currently the only data type with genome-scale coverage among the alternatives, protein or metabolic flux measurements, and (*ii*) transcript are not meant as proportional proxies of metabolic activity, but are rather used to constrain the fluxes in the large -scale model. While such an approach provides the basis for genome-wide differential flux profiling, it faces the challenge of multiple alternative optima whereby metabolic predictions for the same context-specific data^31^, i.e., different metabolic states equally fit the data. Therefore, a robust differential analysis between cell-specific metabolic states requires *a priori* evaluation of the alternative optima, as to avoid biased conclusions based on selecting a single optimal metabolic state as a representative.

The main contributions from our constraint-based modeling study based on integration of G- and M-specific transcriptomics data include the following: (*i*) anaplerotic carbon fixation by phosphoenolpyruvate carboxylase (PEPc) is an important contributor to the production of malate in G cells, (*ii*) transport of oxaloacetate (OAA) to the mitochondria followed by malate production and its export to the cytosol is the main contributor to the cytosolic malate pool, (*iii*) G cells perform an active photophosphorylation comparable to that of M cells, but they differ in the production of NADPH, and (*iv*) sucrose synthesis is dominant in G cells due to the presence of a futile cycle, not due to starch degradation. Our results suggested that G cells have adapted their metabolism towards production of malate and NADPH. We then showed that the key modeling predictions were robust and were in line with data from an independent ^13^C labeling experiment, performed under similar conditions to those of the transcriptomic data. Therefore, our study constitutes a first step towards a quantitative, genome-scale analysis of the metabolic adaptations of G cells, and paves the way to further extensions to obtain a complete understanding of G cell physiology, with possible applications to crop engineering.

## Results and Discussion

### Computational workflow and rationale for model-driven predictions of differences in G and M cell metabolism

To arrive at cell-specific metabolic predictions, we integrated G- and M-specific gene expression data in a modified version of the AraCORE model^17^, here named AraCOREred (Material & Methods, Supplementary Figure S1, Appendix S1, Supplementary File S1). Our approach is fundamentally data-driven, and we did not include any cell-specific metabolic constraints to avoid bias while minimizing the discordance between fluxes and associated transcript levels. In constraint-based metabolic modeling, a metabolic state is characterized by the steady-state flux values through the reactions in the system^32^. In addition, a metabolite can be described by the sum of steady-state fluxes of reactions in which the metabolite participates. This sum of fluxes is referred to as a flux-sum^33^, and quantifies the flux through the pool of the respective metabolite. Therefore, here we predicted reaction fluxes and metabolite flux-sums from flux distributions in concordance with the data, which we then employed to dissect the differences between G and M cell metabolism.

Since the optimum to this multidimensional optimization problem is often not unique, we considered the set of alternative optima to this minimization problem, *i*.*e*., the set of flux distributions that are equally similar to the data. To this end, we first obtained a representative sample of the steady-state reaction fluxes and metabolite flux-sums from the optimal solution space. We then applied a Mann-Whitney test to the resulting distributions to assess if, in a given cell type, a particular reaction or a metabolite showed significantly greater flux or flux-sum value, respectively. In addition, we used a complementary approach in which the extreme alternative optimal flux values for each reaction in AraCOREred—*i*.*e*., minimum and maximum flux values equally concordant with data—were computed and compared between G and M cells. We stress that a comparison of alternative optimal samples of flux values is preferred over a comparison of extreme flux values at alternative optima. The reason is that the flux range alone is not sufficient to provide a robust comparison between the metabolic states of the two cell types. In fact, it may be the case for a reaction to have the same minimum and maximum alternative optimal flux values in both cell types, whereas the distribution of flux values can markedly differ. For instance, this is the case if in one cell type the distribution is skewed to the minimum flux value and, in the other cell type, to the maximum.

To facilitate the interpretation of the predictions, we also determined and reported the mean flux and flux-sum values of each distribution and their ratios between G and M cells (all flux and flux-sum values are expressed in arbitrary units). However, we would like to stress that the differential analysis of fluxes is based on comparison of distributions of data-compatible flux values, by employing the Mann-Whitney test, and not on the comparison of their means. Therefore, in this study we refer to a flux as differential if its respective distributions differ, although these distributions may have the same mean.

### Interplay between the tricarboxylic acid (TCA) cycle and PEPc in the synthesis of cytosolic malate

Malate has been repeatedly identified as a major osmoregulator controlling stomatal opening and closure^4, 34^. Cytosolic carbon fixation by PEPc, followed by reduction of oxaloacetate (OAA) by cytosolic NADP-dependent malate dehydrogenase (NADP-MDH) may represent an additional source of malate in G cells, supported by ^14^C and ^13^C labeling experiments^35–38^ and by gene expression and enzyme activity measurements^28–30, 39, 40^. These studies showed a high expression or activity of enzymes of anaplerotic fixation pathway in G cells^41^. However, these findings have been countered by others claiming that malate content in G cells primarily depends on the supply from the surrounding M cells^42–44^. Therefore, although there is a general consensus in considering malate a key regulator of stomatal regulation, its source in G cells remains unclear.

To delineate which pathway was the main contributor to shaping the pool of malate, we analyzed the relative contributions of cytosolic PEPc – NADP-MDH and the TCA cycle to malate production in G and M cells. This modeling strategy avoids setting a lower bound on non-zero malate uptake (from M cells) and allows an unbiased comparison of fluxes in the two cell types.

**Figure 1:**
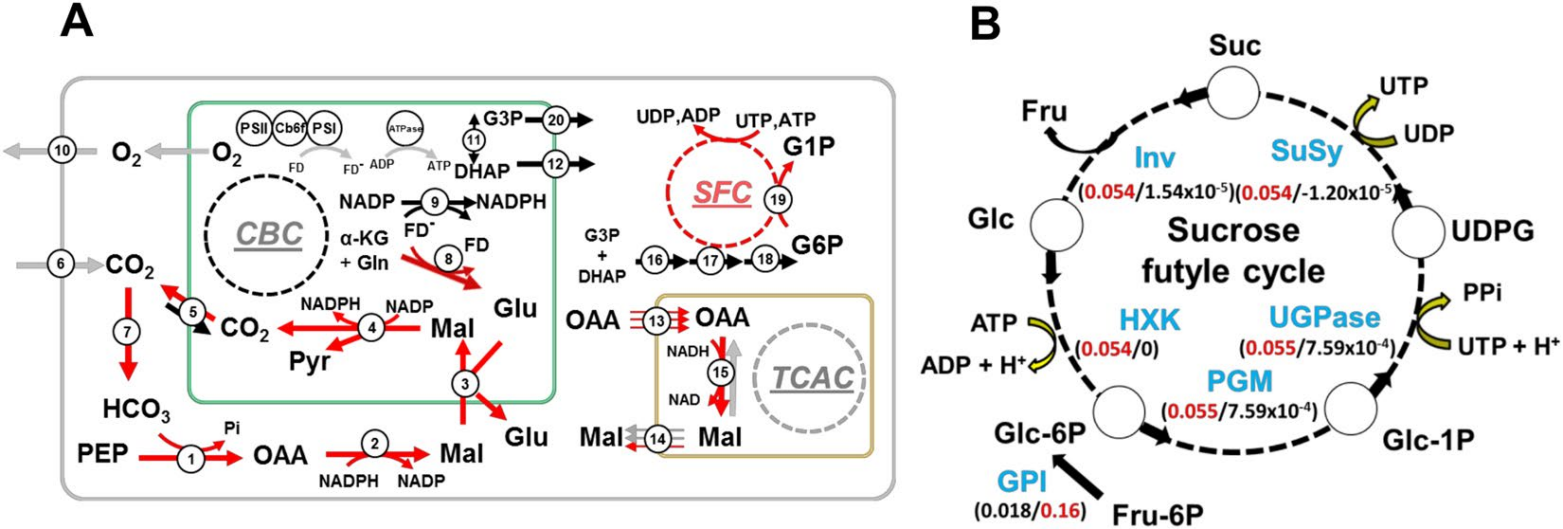
(**A**) A comparison of the predicted metabolic state of G and M cells and (**B**) detailed depiction of the sucrose futile cycle (SFC) predicted to take place in G cells. (**A**) Reactions colored in red (resp. black) carry significantly larger mean flux values in G (resp. M) cells. Reactions depicted in gray cannot be discriminated in terms of mean flux values between the two cell types. The numbers on the reactions correspond to the indices in Supplementary Table S1. The abbreviations used in this figure correspond to: PEP, Phosphoenolpyruvate, Pyr, Pyruvate, Mal, Malate, OAA, Oxaloacetate, Glu, Glutamate, Gln, Glutamine, α – KG, α – Ketoglutarate, FD, Ferredoxin, FD^−^, reduced Ferredoxin, DHAP, Dihydroxyacetone phosphate, G3P, Glyceraldehyde 3-phosphate, G1P, Glucose 1-phosphate, G6P, Glucose 6-phosphate. (**B**) This cycle is composed by five reactions in which sucrose is preferentially degraded into glucose and fructose by the activity of invertase (Inv, index number 45 in AraCOREred) and resynthesized following activities of hexokinase (HXK, index number 31), phosphoglucomutase (PGM, index number 40), UDP-glucosepyrophosphorylase (UGPase, index number 41) and sucrose synthase (SuSy). In M cells, Glucose 6-phosphate was synthetized exclusively by the action of Glucose 6-phosphate isomerase (GPI, index number 39). Values in parenthesis correspond to the predicted mean flux values for each reaction, values in red correspond to G cells while values in black to M cells. A detailed comparison of the flux values for the reactions in the CBC is provided in Table S3.

In comparison to M cells, the mean fluxes in G cells through the triad: Carbonic anhydrase (CA), PEPc and cytosolic NADP-MDH, were upregulated by a factor of ~12, 12 and 2, respectively, that was significant when comparing the distributions of alternative optimal flux values (Fig. 1A and Supplementary Table S1). These predictions demonstrated that the anaplerotic CO_2_ fixation by PEPc plays a significant role in the production of malate in G cells. Since the average increase in the flux through NADP-MDH was smaller than the production of OAA by PEPc, it could be that a great part of the produced OAA is exported to the mitochondria and then converted into malate by the activity of NAD-MDH. To test this possibility, we next inspected the contribution of the TCA cycle to the production of cytosolic malate in G and M cells.

In both G and M cells, model simulations predicted a net transport of OAA to the mitochondrion—through three citrate-, isocitrate- and *cis*-aconitate-dependent antiporters— followed by malate production through NAD-MDH and an export of malate back to cytosol (Fig. 1A). Further, this pathway was predicted to be the main contributor to the total pool of cytosolic malate in both G and M cells. Specifically, malate was exported out of the mitochondrion with an averaged flux value of 0.311, which constitutes a ~2-fold increase as compared to the mean flux value through the cytosolic NAD-MDH in G cells (Fig. 1A and Supplementary Table S1). In the case of M cells, the flux through the malate antiporters, averaging to 0.292, was ~3.5 larger than the cytosolic counterpart, which had a mean flux value of 0.084 (Fig. 1A and Supplementary Table S1). Finally, the flux values through the mitochondrial production and export of malate were all significantly larger in G cells (p-value < 1.19 × 10^−6^, one-sided Mann-Whitney test, Supplementary Table S1), although the differences were slight—the maximum fold change, ~1.2, corresponded to the mitochondrial NAD-MDH.

Taken together, our predictions suggested that both PEPc – NADP-MDH and the TCA cycle are important contributors to malate synthesis in G cells, although the TCA cycle was the main contributor to the pool. In addition, the marked increment in cytosolic malate production in G cells suggested a diverting pathway to reallocate the excess of cytosolic malate in G cells, especially since mitochondrial malate production was almost the same in the two cell types. This was confirmed by significantly larger flux-sums values of malate in G cells in comparison to M cells, particularly for the case of chloroplasts with a mean flux-sum value in G cells of 2.211 in comparison to 1.493 in M cells (Supplementary Table S2). In fact, model predictions showed a marked 7.5-fold increment in the transport of cytosolic malate to chloroplast in G cells (Fig. 1A and Supplementary Table S1).

### Chloroplasts adapt their function to meet the metabolic requirements of G cells

Despite decades of research, the role of chloroplasts in G cells and their potential in providing energy for stomatal adjustments or coordination of redox potential is still unresolved. G cells appear to be highly specialized for solute accumulation and are well equipped to generate the energy required for the uptake of ions (*e*.*g*. K^+^, Cl^−^), synthesis of organic anions (particularly malate^2−^) and accumulation of osmotically active sugars, such as sucrose^45, 46^. Moreover, G cells are known to have fewer and smaller chloroplasts^47^ and lower levels of chlorophyll and ribulose-1,5-biphosphate carboxylase/oxygenase (RuBisCO) compared to M cells^37, 48^. Therefore, we hypothesized that G cell chloroplasts are adapted to meet the specific metabolic requirements needed for stomatal functioning, rather than accomplishing the typical tasks of photosynthetic carbon fixation of M cells, *i*.*e*. to produce sucrose and starch.

To test this hypothesis, we first analyzed the light-dependent reactions in the models specific to G and M cells. We found no significant differences in flux values, neither across the electron transport chain (*i*.*e*. photosystem II, cytochrome b_6_f and photosystem I) nor through the chloroplast ATPase (Fig. 1A and Supplementary Table S1). Thus, G cells were predicted to conduct an active photophosphorylation, which was comparable in magnitude to that of M cells. In fact, the flux-sums of ATP in chloroplast were identical in both cell-types (Supplementary Table S2). This result is in agreement with previous observations^49, 50^, since it has been shown that chloroplasts of G cells are an important source of ATP and NADPH^51, 52^ and are essential for blue-light induced stomatal opening^53^.

However, G and M cells produced NADPH differently. Almost all plastidial NADPH was obtained in M cells through the canonical ferredoxin-NADP reductase, which carried a mean flux value of 0.25. In contrast, in G cells, the mean flux value through this reaction was halved (with a flux of 0.126), even though the production of reduced ferredoxin was indistinguishable in both cell types (Fig. 1A and Supplementary Table 1). The remaining reduced ferredoxin was predicted to be involved in glutamate synthesis in G cells, through the ferredoxin-dependent glutamate synthase, which carried a mean flux value of 0.124. In contrast, the mean flux value in M cells was only 3.671 × 10^−5^ (Fig. 1A and Supplementary Table S1). The rest of the NADPH in G cells was generated by malate decarboxylation in chloroplasts of G cells by the malic enzyme, to compensate for the lower activity of the ferredoxin-NADP reductase. Interestingly, early studies pointed at malate decarboxylation by malic enzyme as playing a key role in guard cell functioning^4^. Moreover, glutamate in chloroplasts of G cells was transported to cytosol in exchange of cytosolic malate by the dicarboxylate transporter. The flux through the latter was predicted to be 7.5-fold larger in G cells, with mean flux values of 0.251 versus 0.033, respectively (Fig. 1A and Supplementary Table S1).

Finally, the flux through CO_2_ diffusion from chloroplast to cytosol was 17-fold larger in G cells, with mean flux values of 0.230 in G cells and 0.013 in M cells (Fig. 1A and Supplementary Table 1). This result largely corresponded to the excess of CO_2_ from malate decarboxylation that was not fixed by RuBisCO. Moreover, our predictions indicated that the exported CO_2_ was largely re-fixed by PEPc in the cytosol. These claims can be made since the model incorporates the diffusion of CO_2_ to and from the environment, between cellular compartments, as well as the interconversion of CO_2_ into bicarbonate (HCO_3_). Taken together, these reactions formed a cycle in G cells, where the CO_2_ fixed in cytosol was transported as malate to chloroplasts and partly returned to cytosol after malate decarboxylation, with a net production of NADPH (Fig. 1A and Supplementary Table S1). These results highlight the adaptation of G cells metabolism to produce malate and NADPH given the lower concentration of chlorophyll and RuBisCO found in these cells^47^.

### The Calvin-Benson cycle drives sucrose and starch syntheses in guard cells

Several studies have suggested that sucrose acts as an important regulator in G cells and, thus, plays a key role in stomatal movement^41^. However, the extent to which G cells are able to produce sucrose on their own is a point of debate. On the one hand, due to the low rate of CO_2_ fixation, it has been suggested that the contribution of the Calvin-Benson cycle (CBC) to sucrose synthesis in this cell type is negligible. On the other hand, other studies have identified scenarios in which the CBC exhibits a significant activity in G cells^54, 55^. Moreover, C fixation by PEPc has been proposed as another route to incorporate C skeletons, which could further be used to drive starch and sucrose synthesis via gluconeogenesis^35, 39, 40^. A recent study revealed that G cells can fix CO_2_ by both RuBisCO and PEPc^38^; however, the extent to which each pathway contributes to the overall amount of sucrose remains an open question.

To resolve the controversy, we investigated the metabolic pathways involved in sucrose synthesis in both G and M cells in our modeling framework. Our results showed that the CBC is active in both cell types. However, most of the reactions involved in the CBC had significantly larger distributions of alternative optimal flux values in M cells, with the notable exception of the PGA kinase, with a mean flux value 1.8-fold larger in G cells (Supplementary Table S3). Our predictions indicated that sucrose synthesis was ultimately driven in both scenarios by the CBC through the canonical pathway of exporting plastidial dihydroxyacetone phosphate (DHAP) and glyceraldehyde 3-phosphate (G3P) to the cytosol, followed by the synthesis of fructose, 1,6-bisphosphate. Interestingly, the model predicted that sucrose synthesis was dominant in G cells, supported by a mean flux value of 0.055 through the sucrose synthase (SuSy) in comparison to 1.5410^−5^ in M cells (Fig. 1B). The higher flux through SuSy in G cells was maintained through a futile cycle composed by five reactions (Fig. 1B). Futile cycles are metabolic reactions in which the net energy balance or the carbon flux around is zero or near to it^56^, and are known to occur around sucrose in both sink and source tissues^57, 58^. In our case, sucrose was re-synthesized from UDP-glucose by activity of SuSy, following the activities of invertase (Inv), hexokinase (HXK), phosphoglucomutase (PGM) and UDP-Glucose pyrophosphorylase (UGPase). These reactions resulted in an equal net consumption and production of UTP and H^+^ (Fig. 1B). In fact, the marked differences in sucrose flux-sums between G and M cells (mean flux-sum value of 0.11 in G cells in comparison to 5.57 × 10^−5^ in M cells) were due to the contribution of this futile cycle in G cells (Supplementary Table S2).

Although sucrose synthesis and cleavage must be dynamic processes to control stomatal movement, our predictions resulted from invoking the steady-state assumption. Therefore, we interpreted the previously described sucrose futile cycle as the closest steady-state solution to an underlying dynamical process, in which the synthetic and depleting branches alternate in accord with stomatal movements. Given the high number of mitochondria^47^, the large catabolic activity found in G cells^59^ and the importance of osmolyte accumulation in this cell type^60^, the identified futile cycle could represent a mechanism that allows avoidance of excess starch synthesis. As a result, carbon skeletons are maintained in sucrose and hexoses, rather than starch, and can be readily used as substrate for glycolysis and mitochondrial metabolism.

Given that the CBC was predicted to be active in G cells, we investigated whether it also drives starch synthesis. We found that G cells were predicted to conduct starch synthesis, although fluxes were significantly higher in M cells in two of the total three reactions involved (Supplementary Table S3). Mean flux values throughout starch degradation were in general small in both cell-types, although this process was significantly pronounced in M cells. For instance, mean flux values through the amylase were ~8-fold larger in M cells, and the disproportionating enzyme was predicted to be active only in M cells (Supplementary Table S3). These results suggested that starch degradation was not a major player in sucrose synthesis in G cells.

We also found marked differences between G and M cells in the main source of CO_2_ entering the CBC. Cytosolic CO_2_ diffusion to chloroplast was only present in M cells (mean flux value of 0.01, Fig. 1A and Supplementary Table S1). Conversely, cytosolic malate import to chloroplast by the dicarboxylate transporter, followed by decarboxylation by plastidial MDH, was the main source of CO_2_ in G cells (Fig. 1A and Supplementary Table S1). In addition, as commented in the previous section, cytosolic PEPc was key to driving malate synthesis in G cells and its import to chloroplast. Altogether, these results match the experimental observations from ref. ^38^ and suggest that both, carbon fixation through CBC and cytosolic PEPc followed by gluconeogenesis play a major role in driving sucrose synthesis in G cells. As a result, the findings in ref. 38 serve as validation of our approach to integrating transcriptomics data for the purpose of comparing the distribution of values for particular fluxes at alternative optima. Moreover, they indicate the presence of a C_4_-like metabolism in G cells, in which the CO_2_ fixation by RuBisCO is derived from decarboxylation of the C_4_ acid malate.

### Robustness of prediction to adding constraints derived from experimental observations

Our computational results presented were generated by constraining the fluxes with G cell- and M-specific expression data. Therefore, no assumptions about the activity of particular reactions were considered—besides imposing a minimal flux through biomass production and the energy maintenance reactions—as to avoid biased predictions. However, we observed two modeling predictions that were unlikely under the photosynthetically active scenario evaluated here. In the first case, RuBisCO oxygenation was absent in both G and M cells, while experimental evidence constraints the ratio of RuBisCO’s carboxylation to oxygenation to be within 1.5 and 4 for both cell types^61–63^. In the second case, three reactions in the CBC: Fructose,1,6-Bisphosphatase, sedoheptulose 1,7-bisphosphate aldolase and sedoheptulose-1,7-bisphosphatase (reaction numbers 11, 13 and 14 in AraCOREred) carried very low or no flux values, thus compromising the functional integrity of the CBC.

To address these inconsistencies, we repeated the computational analysis adding this time a constraint on the carboxylation to oxygenation ratio and including a minimum flux value through the three mentioned reactions in the CBC (Materials & Methods). We next evaluated the qualitative changes upon the inclusion of these additional constraints on the main computational results previously generated. To this end, we looked at the differences in the outcomes of the Mann-Whitney tests—comparing the fluxes through each reaction in G and M cells—between the modeling predictions when no additional constraint was considered and upon the inclusion of the carboxylation to oxygenation and the minimum flux value constraints discussed above. We found that 26.67% of the reactions in AraCOREred changed the Mann-Whitney test status when including the carboxylation to oxygenation constraint and the minimum flux value constraints through the CBC (this figure was reduced to 26.1% when reactions in the CBC, directly affected by the imposed constraints, were not taken into account). However, the vast majority of these changes did not qualitatively affect the main results presented in this study. For instance, the G/M mean flux ratio through the triad CA, PEPc and cytosolic NADP-MDH, shifted from ~12, ~12 and ~2 to ~37, ~37 and ~2, and the mean flux ratios through the ferredoxin NADP-reductase and the glutamate synthase shifted from 0.502 to 0.520 and from ~3391 to ~719, respectively (Supplementary Tables S6, S7 and S8 display the full list for comparison). Therefore, our main results are robust upon the inclusion of these additional, biologically relevant, constraints.

### Validation of model predictions

In this section, we provide a description of the findings from an independent gas chromatography mass spectrometry (GCMS)-based ^13^C-labelling experiment which we employed to validate the flux-based predictions. The employed GCMS approach does not allow us to analyse the ^13^C flux distribution in intermediates from CBC and glycolysis. Therefore, we focused the analysis on sucrose and metabolites related to photorespiration, amino acid metabolism, anaplerotic CO_2_ fixation and the TCA cycle.

### Guard cells have higher anaplerotic CO_2_ fixation

The anaplerotic reaction catalysed by PEPc is characterized by the incorporation of a molecule of HCO_3_ into PEP producing OAA and Pi^64^. The C fixed by PEPc is incorporated in the fourth C of OAA^65^, which can be directly converted to Asp or malate by aspartate amino transferase (AspAT) or MDH, respectively. The anaplerotic CO_2_ fixation is the main source of C incorporation in cells with C_4_ or CAM metabolism, in contrast to C_3_ cells^66, 67^. It has been hypothesized that the anaplerotic CO_2_ fixation by PEPc activity is higher in G cells in comparison to M cells^37, 38, 46^. This idea is supported by the higher expression of genes related to this pathway in G cells in comparison to M cells^24, 27–29^. Recent results from a ^13^C-isotope labelling study strongly suggest that G cells are able to fix CO_2_ by both pathways those catalysed by RuBisCO and PEPc^38^. However, despite the evidences pointing for a differential anaplerotic activity in G cells, this hypothesis has not yet been adequately tested.

**Figure 2:**
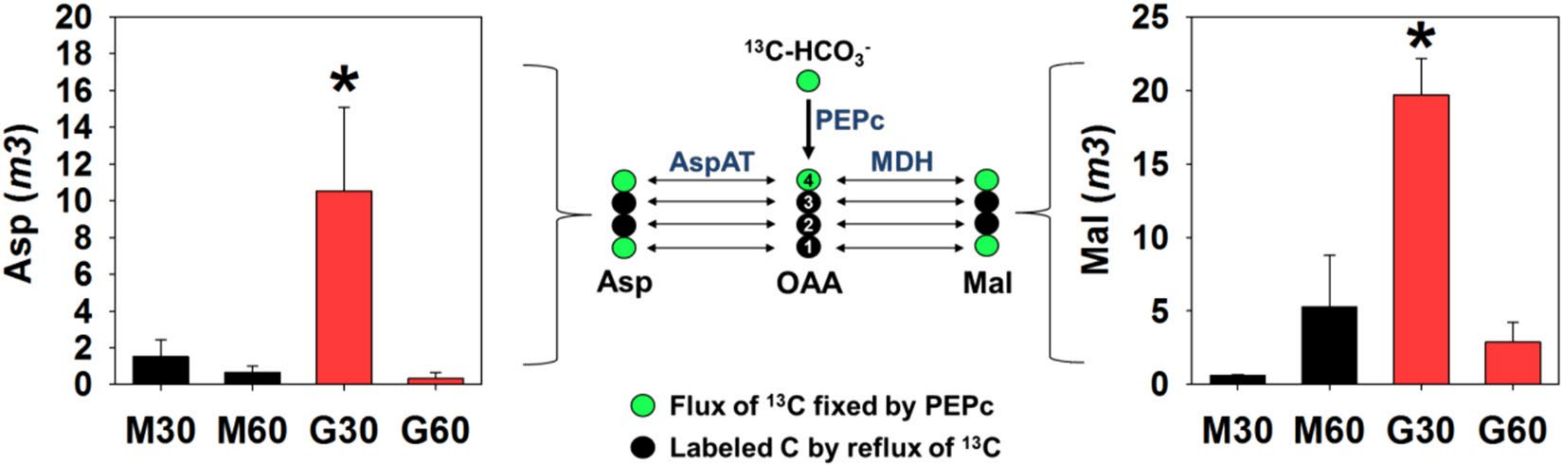
Evidence for the higher anaplerotic CO2 fixation in G cells in comparison to M cells. M cells (black bars) and G cells (red bars) were fed with ^13^-NaHCO_3_ and harvested after 30 min and 60 min in the light. The abundance of mass isotopomers of aspartate *m3* (left side) and malate *m3* (right side) in mesophyll cells (M) or guard cells (G) after 30 and 60 min in the light is displayed. The anaplerotic reaction catalysed by phospho*enol*pyruvate carboxylase (PEPc) and the subsequent steps catalysed by aspartate aminotransferase (AspAT) and malate dehydrogenase (MDH) are highlighted in the center of the figure. Small spheres represent carbon atoms labelled directly by the activity of PEPc (green spheres) or by the reflux of this ^13^C by the activity of the tricarboxylic acid cycle (black spheres). Asterisks indicate values that are significantly different between mesophyll and guard cells by Student’s *t*-test (*P* < 0.05) in the same time point. Data presented are mean ± standard deviation (*n* = 3).

**Figure 3:**
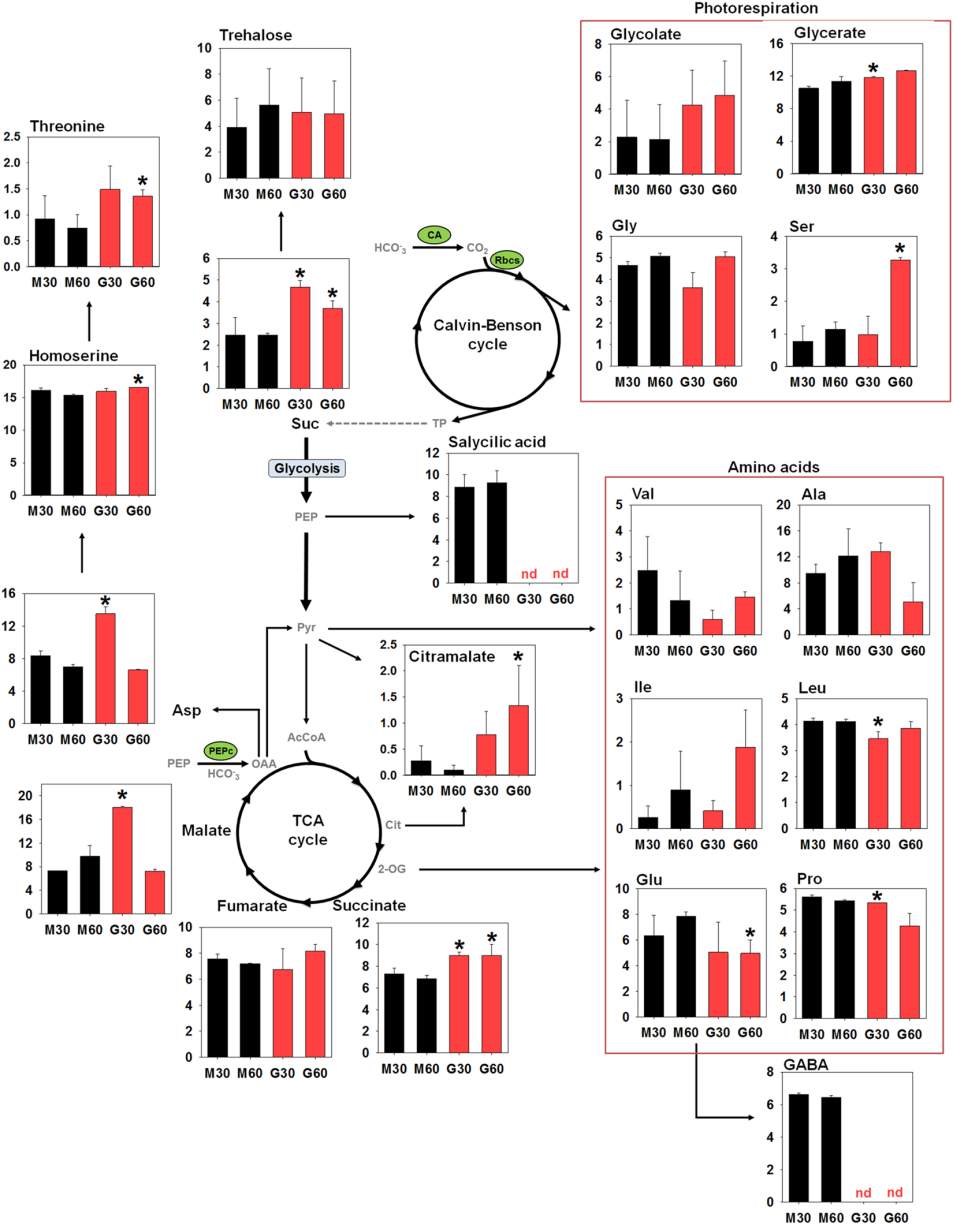
^13^C-enrichment in primary metabolites. M cells (black bars) and G cells (red bars) were fed with ^13^-NaHCO_3_ and harvested after 30 min and 60 min in the light. Asterisks indicate values that are significantly different between M and G cells, (Student’s *t*-test, *P* < 0.05) for the same time point. Data presented are mean ± standard deviation (*n* = 3). The complete list of the ^13^C-enrichment is presented in Supplementary Table S11. Abbreviations: metabolites: GABA, gamma-aminobutyric acid; Suc, sucrose. Enzymes: CA, carbonic anhydrase; PEPc, phospho*enol*pyruvate carboxylase; Rbcs, ribulose-1,5-biphosphhate carboxylase/oxygenase. Amino acids are abbreviated using the standard three-letters code.

Here, we used ^13^C-isotope labelling approach to validate the predictions about anaplerotic CO_2_ fixation. Given the instability of OAA in GCMS-based analysis, we focused on the malate and Asp given that these metabolites are primary products of OAA conversion. The relative isotopomer analysis revealed that the full labelled ion (*m3*) of malate and Asp was ~34- and ~7-fold higher in G than M cells after 30 min under light, respectively (Fig. 2). The level of the fully labelled ion of Asp and malate decreased from 30 to 60 min under light, suggesting that these metabolites are degraded or exported out of G cells. Although we cannot exclude the possibility of efflux of these metabolites from G cells, they can also be employed to increase the flux through the TCA cycle. This idea is supported by the increase in the ^13^C-enrichment in citramalate, succinate and (to a lower extent) fumarate after 60 min under light (Fig. 3).

The higher level of Asp and malate *m3* led to a higher ^13^C-enrichment in these metabolites in G cells in comparison to M cells (Fig. 3). In analyses that take into account the concentration of the metabolites, we also found higher percentage (%) and total ^13^C-enrichment in Asp and malate in G cells (Tables S9 and S10). The fully labelled malate is not only due the PEPc activity, but it also depends on labelled C from glycolysis and the TCA cycle. As stated above, PEPc fixes CO_2_ onto the fourth C of OAA, which can be then converted to malate, producing malate with maximum of two ^13^C (refer to green spheres on Fig. 2). Therefore, the other ^13^C detected in malate and Asp obligatorily comes from fully labelled Acetyl-CoA, which is derived from glycolysis and its assimilation provides two additional ^13^C to metabolites of, or associated to, the TCA cycle^38^. These results were in line with the predictions about larger flux-sums of malate in G in comparison to M cells (Supplementary Table S2). Further, G cells showed higher ^13^C-enrichment in metabolites that can be derived from Asp (*e*.*g*. homoserine and threonine) and malate (*e*.*g*. succinate and citramalate) (Fig. 3 and Supplementary Table S11). Altogether, these results confirmed the modelling predictions and reveal that the anaplerotic CO_2_ fixation catalysed by PEPc is higher in G cells.

### Guard cells have higher ^13^C-enrichment but lower capacity to produce sucrose under ^13^C-NaHCO^−^_3_

Sucrose is the main metabolite translocated throughout the plant and performs several functions in the metabolism^68^. Sucrose can be produced by using triose phosphates and hexoses exported from chloroplast following photosynthesis and starch degradation, respectively^69^, as well as by PEPc fixation and gluconeogenesis^70^. The capacity of G cells to produce sufficient quantity of sucrose has long been debated. Under the experimental condition used here, we observed higher ^13^C-enrichment in sucrose after ^13^C-NaHCO^−^_3_ incorporation in G cells (Fig. 3), which confirms the predictions of the model (Fig. 1A,B). However, it is important to note that M cells had, on average, 2.2-fold more sucrose than G cells (Supplementary Table S12). The difference in the amounts of sucrose between the two cell types leads to an equal percentage of ^13^C-enrichment and total ^13^C-enrichment in sucrose between G and M cells, with both calculations taking into account the amount of the metabolite of each cell type (Tables S9 and S10). Moreover, the higher ^13^C-enrichment observed in G cells (Fig. 3) may be due the use of HCO_3_ as labelled substrate, which could favour the fixation by PEPc rather than by RuBisCO. The higher expression of CA^24, 30^ and higher malate decarboxylation in the chloroplast of G cells, as predicted by the model, may create a high CO_2_-concentrated atmosphere around RuBisCO of G cells, similarly to what has been observed in C_4_ cells. This would optimize the plastidial CO_2_ fixation by RuBisCO in these cells, leading to higher ^13^C-enrichment in sucrose. This idea is further supported by the higher ^13^C-enrichment observed in metabolites from photorespiratory pathway such as Ser and glycerate (Fig. 3 and Supplementary Table S11). However, further experimental evidence is needed to confirm this hypothesis and the model predictions.

Sucrose was thought to act as an osmolyte for G cell regulation^71^. However, recent evidences suggest that the role of sucrose for G cells regulation may be primarily energetic^38, 72–74^ and that sugar metabolism and HXK activity may be pivotal in the control of stomatal movements^75, 76^. The model used here predicted that higher fluxes through sucrose occur in G cells and this can be explained by a substrate (futile) cycle formed around this metabolite. Substrate cycles have been proposed to be important for the regulation of plant metabolism^58, 77–81^, despite their “futile” designation. It is known that sucrose and hexose cycles are supported by high activity of enzymes such as SuSy, Inv, HXK, sucrose-phosphate synthase (SPS) and other sugar-related enzymes^80, 82, 83^. Interestingly, most of these enzymes are highly expressed in G cells^41^, in further support for the idea of the substrate cycle predicted by the model. Additionally, futile cycles have been confirmed *in vivo* by steady-state and pulse-labelling approaches using both ^14^C and ^13^C substrates^81^, which can be used and are required to confirm our model predictions.

## Conclusions

Despite decades of research, the role of central carbon metabolism on the functions of G cells remains poorly understood. Here, we used transcriptomics data and a large-scale metabolic model to predict pathways with differential flux profiles between G and M cells. Our analysis pinpointed reactions whose distributions of fluxes in the space of alternative optima differ between G and M cells. Since reaction fluxes are difficult to be experimentally estimated in photoautotrophic growth conditions, we predicted flux-sums as descriptors of metabolite turnover and validated the qualitative behavior via an independent ^13^C-labeling experiment. Our results highlighted the metabolic differentiation of G cells as compared to the surrounding M cells, and strengthen the idea of occurrence of a C_4_-like metabolism in G cell, as evidenced by the higher anaplerotic CO_2_ fixation in this cell. Moreover, our modeling approach brings important and new information concerning CBC and sucrose metabolism in G cells, indicating that the main source of CO_2_ for RuBisCO comes from malate decarboxylation rather than CO_2_ diffusion and that G cells have a futile cycle around sucrose. The modeling and data integration strategy can be used in future studies to investigate the concordance between flux estimates with data from different cellular layers. In addition, future studies on guard cell physiology would benefit from coupling the flux-centered genome-scale modeling framework presented in this study with existing kinetic models of stomatal movement, such as OnGuard^9^. Finally, although still technically challenging, future studies would also benefit from quantitative experimental data of coupled G and M cells *in vivo*, which could be integrated in a unified modeling framework addressing the coordination between the two cell types.

## Material and Methods

This section provides the details of the computational methods used in the metabolic modeling. A depiction of the general procedure followed is also available in Figure S1. In addition, all the MATLAB code used to obtain the predictions is provided in File S1.

### Gene expression data

G cell gene expression data was obtained from ref. 28 published under the GEO accession numbers GSM918075, GSM918076 and GSM918077, which correspond to three replicates of the same experiment. Expression data of M cells were obtained from ref. 84, also with three replicates with accession numbers GSM486916, GSM486917, and GSM486918. In both cases, expression data were measured from wild type Col-0 *A*. *thaliana*. Data from G cells set was obtained from plants were grown for 8-10 weeks at 22 °C, and in an 8 hours light/16 hours dark cycle under 150 μmol.m^−2^.s^−1^. In the case of the data set from M cells, plants were grown for 5 weeks at 20/16 °C, and in an 8 hours light/16 hours dark cycle under 120 μmol.m^−2^.s^−1^. CEL files were normalized using the RMA method implemented in the *affy* R package^85^. In addition, probe names were mapped to gene names following the workflow described in ref. 86, where probes mapping to more than one gene name are eliminated.

Expression values were mapped to reactions following the gene-protein-reaction rules and a self-developed MATLAB function, *mapgene2rxn*, which can be found in File S1. Specifically, the conditional relation g_i_ AND g_j_ in a given reaction rule was modeled as the minimum expression value of the two genes, g_i_, g_j_. The conditional relation g_i_ OR g_j_ was modeled as the maximum expression of the two genes. This process was repeated for each of the three replicates in each cell-type (*i*.*e*., G and M cells). The mean and standard deviation among replicates were then calculated for each reaction associated gene expression. Finally, values were scaled to the maximum in each experiment to obtain the final expression data used in this study.

### Metabolic network model

AraCORE, a metabolic network model of the primary metabolism of *A*. *thaliana* developed by ref. 17 was used to reconstruct the metabolic networks specific to G and M cells. The model includes 549 reactions and 407 metabolites assigned to four subcellular compartments. The original AraCORE contains exchange reactions that directly link organelles to the environment (*i*.*e*. circumventing the cytosolic compartment). Therefore, all exchange reactions bypassing the cytosol were removed to avoid biased results. Therefore, here, we used a reduced AraCORE version (AraCOREred), available in File S1 that consists of 455 reactions and 374 metabolites.

### Gene expression integration in the AraCOREred metabolic model

The two context-specific flux distributions (*i*.*e*., corresponding to guard cell and mesophyll) were obtained by integrating the expression data into AraCOREred. To this end, we used the RegrEx method^87^, which performs an optimization process to find a feasible flux distribution (*i*.*e*., satisfying the stoichiometric and thermodynamic constraints dictated by the metabolic model used) that maximizes the concordance to the integrated expression data. In addition, for each cell type, the mapped data were scaled by the respective standard deviations (estimated from the three available replicates. In this way, reactions whose associated data were less consistent among replicates (as quantified by the standard deviation) contributed to a lesser extent to the global similarity between the optimal flux distribution and the integrated expression data. Details about the RegrEx implementation as well as the adaptation used in this study can be found in Appendix S1.

The biomass reaction was forced to be active by imposing that the flux, v_bio_, through the reaction “*light-dependent biomass*” (number 454 in AraCOREred) satisfies the constraint *v*_*bio*_ ≥ 10^−6^. Additionally, the fluxes through the three energy maintenance reactions in AraCOREred were forced to be greater or equal to 0.001. The three maintenance reactions represent the consumption of ATP by non-metabolic processes—*i*.*e*., apart from the consumption in the reactions included in AraCOREred—in the cytosol (reaction index number 448), chloroplast (449) and mitochondrion (450), respectively. The lower bound values were chosen to represent approximately a 20% of the theoretically maximum flux value for each reaction in the alternative optima space of RegrEx (as calculated per RegrEx_FVA_).

### Evaluation of the alternative optima space

A flux distribution leading to an optimal value for the RegrEx objective is likely not unique since typically not all reactions in a genome-scale model are endowed with reaction associated data. Therefore, there are reactions whose flux value can vary without affecting the overall similarity between the flux distribution and the expression data used. This generates an alternative optima space of RegrEx solutions populated by feasible flux distributions that are equally concordant to the expression data being integrated. We investigated the space of alternative RegrEx solutions with two complementary approaches: a sampling algorithm, RegrEx_AOS_ and a FVA-like (for Flux Variability Analysis) to compute the extreme values, RegrEx_FVA_, both algorithms fully described in Appendix S1 and available in MATLAB code in File S1.

RegrEx_AOS_ generates a uniform sample of the alternative optima space of flux distributions, which, in turn, allows analyzing the distribution of flux values that each reaction takes in the alternative optimum space. In this study, we used RegrEx_AOS_ to generate a sample, ${V}_{AO}=\{{v}_{i,k}^{\ast },\,i=1,\ldots ,{N}_{R},\,k=1,\ldots ,n\}$, of *n* = 2 · 10^4^ random alternative optimum flux distributions (containing *N*_*R*_ = 455 reactions) for each of the cell-specific scenarios. On the other hand, RegrEx_FVA_ computes the minimum and maximum allowable flux values in the alternative optima space. To this end, RegrEx_FVA_ adapts the Flux Variability Analysis^88^ procedure, originally designed to investigate the alternative optima space of the linear programs behind Flux Balance Analysis^32^, to the particular computational setup of RegrEx. Supplementary Tables S4 and S5 show the extreme values for the reactions displayed in Supplementary Tables S1 and S3 (a complete list can be found in the MATLAB data file in Supplementary File S1).

### Evaluation of flux values across the alternative optima space

Next, the previously generated distributions of alternative flux values of each reaction were compared between G and M cells. To this end, a Mann–Whitney test^89^ (*ranksum* MATLAB function) was applied to obtain the set of reactions showing significantly increased flux values across the alternative optima space for each cell-type. Specifically, we performed a right-tailed test with null hypothesis stating that there were not differences between the two cell types and alternative hypothesis stating that one cell-type (*i*.*e*., guard cells or mesophyll depending on the comparison) had a bigger flux distribution than the other one, rejecting the null hypothesis at the significance level of α = 0.05. In addition, we performed a two-tailed Mann-Whitney test evaluating only the significance of the difference between two distributions, *i*.*e*., with null hypothesis stating no differences and alternative hypothesis stating significant differences (either larger or smaller) between the two distributions. In this study, only distributions that passed the two tests, *i*.*e*., significantly larger (or smaller) and significantly different, were taken into account, as to prevent inconsistent results.

### Evaluation of flux-sum values across the alternative optima space

We calculated the flux-sum values for each metabolite *m* (*f*_*m*,*k*_) in the AraCOREred model and for each alternative optimal flux distribution, ${v}_{k}^{\ast }\in {V}_{AO}$ and cell-type as follows:

1 $${f}_{m,k}=\sum _{j}|{v}_{j,k}^{\ast }|,j\in {R}_{m}$$

where *R*_*m*_ is the index set corresponding to reactions in which metabolite *m* participates either as a substrate or as a product. This procedure generated a distribution of alternative flux-sum values for each metabolite in each cell-type. Next, the previously generated distributions of flux-sum values of each metabolite were compared between G and M cells. To this end, we applied the same battery of Mann-Whitney tests previously used to compare the distributions of alternative optimal flux values.

In this analysis, the different subcellular localizations of a given metabolite were treated as different metabolites in the metabolic network, due to the compartmentalization of the AraCOREred model—which is subdivided into cytosol, mitochondrion, chloroplast, and peroxisome. Therefore, the distribution of flux-sum values, *f*_*m*,*k*_, presented above, was obtained specifically for each subcellular localization of a given metabolite. However, the metabolic experimental data generated in this study do not discriminate between subcellular localizations of the measured metabolites—*i*.*e*., the data measure the total cellular pool of a metabolite and not the specific concentrations in each subcellular compartment. To match the experimental conditions, we additionally calculated the flux-sums of the metabolites with experimental data across all subcellular compartments. Specifically, in (1), the reaction index set, *R*_*m*,*_, of a metabolite, *m* ^*^, with experimental data, contained all reactions in which *m* ^*^ participated across all subcellular compartments. The same statistical analysis used to compare the flux-sum distributions of compartmentalized metabolites was applied in this case.

### Integration of additional constraints derived from experimental observations

Bounds on the carboxylation to oxygenation ratio of RuBisCO were included in the following way. Let *v*_*C*_ denote the flux through RuBisCO carboxylation and *v*_*O*_ that of the oxygenation, then the non-linear constraint

2 $${r}_{lb}\le \,\frac{{v}_{C}}{{v}_{O}}\le {r}_{ub}$$

can be transformed into the pair of linear constraints

3 $${r}_{lb}{v}_{O}-{v}_{C}\le 0$$

4 $${r}_{ub}{v}_{O}-{v}_{C}\ge 0$$

where *r*_*lb*_, *r*_*ub*_ respectively denote the lower and upper bound of the carboxylation to oxygenation ratio. These linear constraints were integrated as additional constraints to the optimization programs performed by RegrEx_LAD_ and RegrEx_AOS_ (Appendix S1) to guarantee that any alternative optimal solution agreed with the specified bounds (in this study *r*_*lb*_ = 1.5, *r*_*ub*_ = 4. Constraints regarding the minimum flux through the reactions: *Fructose*,*1*,*6-Bisphosphatase*, *sedoheptulose 1*,*7-bisphosphate aldolase* and *sedoheptulose-1*,*7-bisphosphatase* (reaction number 11, 13 and 14 in AraCOREred) where integrated by increasing the lower bound through these reactions from zero to a small amount (0.001 in this study).

To evaluate the changes in the simulation results due to the integration of the new set of constraints, we compared the outcomes of the Mann-Whitney tests across all reactions in the AraCOREred model. Concretely, we first transformed the vector of p-values resulting from the comparison between G and M cells of the distributions of alternative optimal flux values for each reaction, into a binary vector. To this end, p-values below the significance threshold α = 0.05 were mapped to 0, and the rest to 1. This process was repeated for each scenario: (i) the original results without additional constraints, (ii) the results generated after constraining the carboxylation to oxygenation ratio and (iii) the results generated when constraining the carboxylation to oxygenation ratio and the flux through the three above mentioned reactions. We next computed the Hamming distance between the three binary vectors. In this case, we evaluated the distance between the whole set of reactions in the AraCOREred model, and between all reactions except those from the CBC, since reactions in the CBC were directly affected by the newly imposed constraints.

### Plant material and growth conditions

Seeds of wild type *Arabidopsis thaliana* L. plants (Columbia ecotype) were handled as described previously^90^. Fully expanded rosette leaves of 5-week-old plants grown under long day conditions (16 h light/8 h dark), light intensity 100 µmol photons m^−2^ s^−1^ and temperature 20 °C ± 2 were harvested for isolation of both G cells and mesophyll cell protoplasts (MCP).

### Experimental set-up for *in vivo* guard cell and mesophyll cell analyses

We recently developed a methodology to perform ^13^C kinetic isotope labeling experiments in isolated G cell enriched epidermal fragments^38^. Here, we modified this method to analyze the metabolic flux distribution in simultaneously isolated G cells and MCP. Several experiments were performed to simultaneously isolate both cell types from the same plant material as well as to perform a ^13^C kinetic isotope labeling experiment following the metabolic fate of ^13^C-NaHCO_3_ by gas chromatography-time of flight-mass spectrometry. All the solutions used for G cells and MCP isolation were prepared in deionized water and filtered through a 0.45 µm filter. The isolation of both cell types was carried out in the dark using leaves from dark-adapted plants in order to avoid light induced metabolic changes during the isolation of both cell types. Furthermore, in contrast to the original protocols in which the isolated cells are subjected to a high concentration of mannitol (0.4-0.56 M), we decided to reduce the mannitol concentration to minimize the excess of this metabolite in the final steps of the protocol, since this causes problems in subsequent metabolite determination Thus, the concentration of this osmolyte in the medium was reduced gradually from 0.4 M (solution I) to a final concentration of 0.05 M (solution IV - see below). The solutions used for GC and MCP isolation were: **enzymatic solution** - 20 mMMes/NaOH, pH 5.7, 0.4 M mannitol, 10 mM CaCl_2_, 20 mMKCl, 0.1% (w/v) bovine serum albumin (BSA), 1% (w/v) cellulase, Onozuka R10 (Yakult Pharmaceutical Industry Co., Tokyo, Japan), 0.25% (w/v) macerozyme, Onozuka R10 (Yakult Pharmaceutical Industry Co., Tokyo, Japan). **Solution I** - 0.4 M mannitol, 1 mM CaCl_2_.**Solution II** - 20 mMMes/NaOH, pH 6.5, 0.1 M mannitol, 1 mM CaCl_2_. **Solution III** - 20 mMMes/NaOH, pH 6.5, 0.05 M mannitol, 1 mM CaCl_2_, 5 mMKCl and **Solution IV** - 20 mMMes/NaOH, pH 6.5, 0.05 M mannitol, 1 mM CaCl_2_, 5 mMKCl, 1 mM ^13^C-NaHCO_3_.

### ^13^C isotope labelling experiment using isolated mesophyll cell protoplasts

Arabidopsis MCP were isolated from dark adapted five-week-old plants using the TAPE-sandwich method^91^ with modifications. Approximately 20 leaves per replicate were peeled and placed in a petri dish containing 50 mL of enzymatic solution and shaken in the dark. After 90 min, the solution containing MCP was transferred to a 50 mL Falcon tube and centrifuged at 100 *g* for 15 min at 4 °C. The supernatant was removed and the pellet containing MCP was gently re-suspended in solution I and kept on ice and in the dark for 30 min. This procedure was repeated by adding and removing the solutions II and III with the same interval on ice and in the dark. After the addition of the solution IV, the MCP were immediately transferred to the light (45 ± 1 µmol photons m^−2^ s^−1^) and harvested after 30 and 60 min. We adjusted a methodology to rapidly collect and frozen MCP in the light following a previous established methodology developed for ^13^C kinetic labelling experiments in Algae^92^ which the MCPs were vacuum concentrated to a glass filter (1.6 µm). This process was carried out under the same light source used. The time spent between the transfer of the MCP from the petri dishes to the glass filter and the subsequently frozen was around 1–2 min.

### ^13^C kinetic isotope labelling experiment in guard cells

Arabidopsis G cells were isolated from five-week-old plants according to previous methods^38, 93^ with minor modifications. Approximately 30 whole rosettes were ground using a commercial blender with an internal filter (Philips, HR 2084, Amsterdam, The Netherlands) containing 300 ml of cold deionized water for 3 min. The internal filter is important to remove the excess of fibers and mesophyll cells^38^. After that, the isolated guard cell enriched epidermal fragments were collected on a 220 μm nylon mesh and rinsed well with distilled water (1.5 L). After drying the excess of water, the G cell enriched epidermal fragments preparation was transferred to the enzymatic solution and kept for 90 min in the dark with shaking^93^. The guard cell enriched epidermal fragments were collected on a 30 μm nylon mesh, rinsed with solution I and kept in 15 mL of this solution for 30 min on ice and in the dark. The osmotic potential of the solution was decreased by adding 15 mL of the solution II and III with an interval of 15 min on ice. After, G cell enriched epidermal fragments were collected on a 30 μm nylon mesh and transferred to 5 mL of solution III and carefully layered on top of 20 mL Histopaque® solution (Histopaque-1077, Sigma Aldrich, St. Louis, USA) in a 50 mL falcon tube. The tube was centrifuged at 200 *g* for 15 min in order to separate GCs from trichomes and other cell debris. The layer of G cells was withdrawn from the interface of the two solutions with a 5 mL pipette, collected on a 30 μm nylon mesh, transferred to a falcon tube containing solution IV and transferred to the light (45 ± 1 μmol m^−2^ s^−1^). After 30 and 60 min under light, G cells were rapidly vacuum concentrated to a glass filter (1.6 µm) as performed for MCP and frozen.

### Extraction and analysis of metabolites by gas chromatography-time of flight-mass spectrometry

The extraction of polar metabolites from G cells and MCP were carried out following an established gas chromatography-time of flight-mass spectrometry based platform^94^ adapted to G cells^38^. In brief, the extraction of the metabolites was carried out using 1000 µL of methanol (100%) at 70 °C for 1 h with constant agitation. 60 µl of Ribitol (0.2 mg/ml stock in dH_2_O) was added as an internal quantitative standard. The extract was centrifuged at 11000 *g* for 10 min, and 600 µL of the supernatant was transferred to another tube, where 500 µL of chloroform (100%) (LC grade, Sigma) and 800 µL of deionized water were added. After vortexing for 10 s, another centrifugation was carried out for 15 min at 2200 *g*. 1300 µL of the (upper) polar phase was collected, transferred to 2.0 ml tubes, and reduced to dryness in a speed vac. The sample derivatization was carried out using *N*-Methyl-*N*-(trimethylsilyl) trifluoroacetamide (MSTFA, CAS 24589-78-4, Macherey& Nagel, Düren, Germany) and methoxyamine hydrochloride (CAS 593-56-6, Sigma, Munich, Germany) dissolved at 20 mg/ mL in pure pyridine (CAS 110-86-1, Merck, Darmstadt, Germany) (Lisec *et al*.^94^). Metabolites were identified by comparison the Golm metabolome database^95^. The analysis of relative abundance of mass isotopomers was carried out using Xcalibur 2.1 software (Thermo Fisher Scientific, Waltham, MA, USA) exactly as described in ref. 38. Absolute levels and percentage and total ^13^C-enrichment of metabolite was determined as described previously^96, 97^.

## Electronic supplementary material

Supplementary Information

S1File
