# Supplementary material for: Resolving the central metabolism of Arabidopsis guard cells
Source: Sci Rep. 2017 Aug 16;7:8307. doi: 10.1038/s41598-017-07132-9 (PMC5559522; doi:10.1038/s41598-017-07132-9)
Supplement: Supplementary file 1 — Supplementary Information [file 41598_2017_7132_MOESM1_ESM.pdf]

Supplementary Information of the research article:

Resolving the central metabolism of Arabidopsis guard cells

Semidán Robaina-Estévez[1*], Danilo de Menezes Daloso[2,3*], Youjun Zhang[2], Alisdair R. Fernie[2], Zoran Nikoloski[1**]

[1]Systems Biology and Mathematical Modeling Group, Max Planck Institute of Molecular Plant Physiology, Potsdam-Golm, Germany, [2]Bioinformatics Group, Institute of Biochemistry and Biology, University of Potsdam, Potsdam-Golm, Germany, [3]Central Metabolism Group, Max Planck Institute of Molecular Plant Physiology, Potsdam-Golm, Germany, [4]Departamento de Bioquímica e Biologia Molecular, Universidade Federal do Ceará, Fortaleza-CE, Brasil

*these authors contributed equally

**corresponding author: nikoloski@mpimp-golm.mpg.de

Contents

Supplementary Tables S1 to S12

Supplementary Figure S1

Supplementary Appendix S1

Table S1. A comparison of the predicted metabolic state of GC and M. The predicted mean flux values corresponding to the reactions depicted in Figure 1 are shown for G and M cells. The p-values shown in this table correspond to a Mann-Whitney test comparing the distributions of flux values of G and M cells (V_G and V_M, respectively). Three different tests were considered for each comparison: (i) we evaluated whether the two distributions differ (null hypothesis Ho: V_G = V_M, (ii) if G had increased flux values in comparison to M cells (Ho: V_M > V_G), and (iii) if M had increased flux values in comparison to G cells (Ho: V_G > V_M). In all three cases, a horizontal bar indicates a failure of the test due to distributions consisting of a fixed value. Reaction names and index numbers in accord with AraCOREred.

Idx. in Fig.1	Reaction Name (Idx in AraCOREred)	Mean Flux G	Mean Flux M	Mean ratio (G/M)	Ho: $V_G = V_M$	Ho: $V_M > V_G$	Ho: $V_G > V_M$	SubSystem
PSII	photosystem II (1)	0.125	0.125	1.000	0.317	0.159	0.841	light reactions
Cb6f	cytochrom b6f complex (2)	0.250	0.250	1.000	0.317	0.159	0.841	light reactions
PSI	photosystem I (3)	0.500	0.500	1.000	0.317	0.159	0.841	light reactions
ATPase	ATPase (5)	0.107	0.107	1.000	0.317	0.159	0.841	light reactions
1	PEP carboxylase (54)	0.420	0.034	12.486	0	0	1.000	gluconeogenesis
2	Cytosolic NADP-MDH (115)	0.161	0.084	1.917	0	0	1.000	pyruvate metabolism
3	Dicarboxylate transporter (339)	0.251	0.033	7.554	0	0	1.000	transport
4	Plastidial NADP-Malic Enzyme (113)	0.249	0.032	7.848	0	0	1.000	pyruvate metabolism
5	CO2 diffusion [Forward] (374)	0	0.001	0	0	1.000	0	transport
5	CO2 diffusion [Backward] (374)	0.230	0.013	17.342	0	0	1.000	transport
6	Import CO2 (413)	0.021	0.021	1.000	0.013	0.994	0.006	import
7	Carbonic anhydrase (152)	0.420	0.034	12.486	0	0	1.000	carbon fixation
8	Glu tamate synthase (FeS-Fd) (179)	0.124	3.671e-05	3.391e+03	0	0	1.000	glutamate synthesis
9	Ferredoxin-NADP reductase (4)	0.126	0.250	0.502	0	1.000	0	light reactions
10	Export O2 (420)	0.020	0.020	1.000	0.847	0.424	0.576	export
11	TP isomerase [Forward] (9)	0.854	0.855	0.999	0	1.000	0	Calvin-Benson cycle, glycolysis
12	TP/Pi translocator [Forward] (328)	0.857	1.000	0.858	0	1.000	0	transport
13	Di-/ri-carboxylate carrier [Forward] (346)	0.348	0.327	1.065	0	0	1.000	transport
13	Di-/ri-carboxylate carrier [Forward] (347)	0.345	0.324	1.065	0	0	1.000	transport
13	Di-/ri-carboxylate carrier [Forward] (348)	0.349	0.324	1.077	0	0	1.000	transport
14	Di-/ri-carboxylate carrier [Backward] (343)	0.311	0.294	1.059	0	0	1.000	transport
14	Di-/ri-carboxylate carrier [Backward] (344)	0.314	0.289	1.088	0	0	1.000	transport
14	Di-/ri-carboxylate carrier [Backward] (345)	0.309	0.292	1.057	2.395e-06	1.198e-06	1.000	transport
15	Mitochondrlial NAD-MDH [Backward] (80)	0.271	0.226	1.201	0	0	1.000	tricarboxylic acid cycle, glyoxylate cycle
15	Mitochondrial NADP-MDH (117)	0.753	0.724	1.041	0	0	1.000	pyruvate metabolism
16	FBP aldolase [Forward] (35)	0.018	0.160	0.112	0	1.000	0	sucrose synthesis, gluconeogenesis, glycolysis
17	FBPase (36)	0.073	0	Inf	0	0	1.000	sucrose synthesis, gluconeogenesis, glycolysis
17	PPi-dep. Phosphofructokinase [Backward] (136)	0	0.693	0	0	1.000	0	pyrophosphate recycling
18	G6P isomerase [Forward] (39)	0.018	0.160	0.112	0	1.000	0	sucrose synthesis, sucrose degradation, gluconeogenesis
19	Phosphoglucomutase [Forward] (40)	0.055	7.590e-04	72.503	0	0	1.000	sucrose synthesis, sucrose degradation
20	TP/Pi translocator [Forward] (327)	0	0	0	-	-	-	transport
20	TP/Pi translocator [Backward] (327)	0.362	0.439	0.825	0	1.000	0	transport

Table S2. Predicted Flux-Sums of selected metabolites in the AraCOREred model. The predicted mean flux-sum values for several metabolites are displayed. In each of the metabolites, the flux-sum values are split into each cellular compartment: cytosol (c), mitochondrion (m), peroxisome (p) and chloroplast (h). In addition, the total flux-sum values (*i.e.*, taking into account all the compartments) are provided. The interpretation of the p-values is similar to that of Table S1. A horizontal bar indicates a failure of the test due to distributions consisting of a fixed value.

Metabolite (compartment)	Mean FluxSum G	Mean FluxSum M	Ratio (G/M)	Ho: $V_G = V_M$	Ho: $V_M > V_G$	Ho: $V_G > V_M$
Total Mal	5.434	4.581	1.186	0	0	1.000
Mal(c)	2.451	2.172	1.128	0	0	1.000
Mal(m)	2.129	2.004	1.062	0	0	1.000
Mal(p)	0	0	-	-	-	1.000
Mal(h)	2.211	1.493	1.481	0	0	1.000
Total Suc	0.109	5.574e-05	1.948e+03	0	0	1.000
Futile Cycle Suc	0.109	1.203e-05	9.019e+03	0	1.000	0
Total OAA	7.460	6.729	1.109	0	0	1.000
OAA(c)	4.063	3.606	1.127	0	0	1.000
OAA(m)	2.083	1.950	1.069	0	0	1.000
OAA(p)	1.320	1.488	0.887	0	1.000	0
OAA(h)	1.713	1.430	1.198	0	0	1.000
Total Pyr	3.571	3.085	1.158	0	0	1.000
Pyr(c)	1.418	1.344	1.055	0	0	1.000
Pyr(h)	0.498	0.139	3.574	0	0	1.000
Pyr(m)	1.656	1.639	1.010	0	0	1.000
Pyr(p)	1.418	1.268	1.118	0	0	1.000
Total PEP	2.896	2.583	1.121	0	0	1.000
PEP(c)	1.880	1.679	1.120	0	0	1.000
PEP(h)	1.536	1.672	0.919	0	1.000	0
G3P(h)	3.626	2.832	1.280	0	0	1.000
G3P(c)	1.837	0.966	1.901	0	0	1.000
Total ATP	1.967	1.609	1.223	0	0	1.000
ATP(h)	1.050	1.050	1.000	0	0	1.000
ATP(c)	6.691	6.234	1.073	0	0	1.000
ATP(m)	3.445	3.349	1.028	0	0	1.000
Total NADP	1.740	1.437	1.211	0	0	1.000
NADP(h)	1.506	1.447	1.041	0	0	1.000
NADP(c)	6.691	6.234	1.073	0	0	1.000
NADP(m)	3.445	3.349	1.028	0	0	1.000
Total NADPH	1.740	1.437	1.211	0	0	1.000
NADPH(h)	1.506	1.447	1.041	0	0	1.000
NADPH(c)	2.781	1.659	1.677	0	0	1.000
NADPH(m)	2.171	1.602	1.355	0	0	1.000
Total CO_2	0.839	0.070	11.998	0	0	1.000
CO_2(h)	0.001	0.002	0.788	0	1.000	0
CO_2(c)	5.434	4.581	1.186	0	0	1.000
CO_2(m)	2.451	2.172	1.128	0	0	1.000

Table S3. Activity of the CBC and starch metabolism in GC and M. The predicted mean flux values of reactions in the CBC cycle and starch metabolism are shown for G and M cells. The interpretation of the p-values is similar to that of Table S1. A horizontal bar indicates a failure of the test due to distributions consisting of a fixed value. Reaction names and index numbers in accord with AraCOREred.

**Reaction [direction](Idx in AraCOREred)**	**Mean Flux G**	**Mean Flux M**	**Mean ratio (G/M)**	**Ho:** $V_G = V_M$	**Ho:** $V_M > V_G$	**Ho:** $V_G > V_M$
CBC						
RuBisCO Carboxylation (6)	0.019	0.020	0.974	0	1.000	0
RuBisCO Oxygenation(85)	2.043e-06	2.043e-06	1.000	0	0	1.000
PGA kinase [Forward] (7)	0.495	0.278	1.779	0	0	1.000
GAP dehydrogenase (8)	0.864	0.959	0.901	0	1.000	0
TP isomerase [Forward] (9)	0.854	0.855	0.999	0	1.000	0
FBP aldolase [Forward] (10)	0	0	0	-	-	-
FBPase (11)	0	0	0	-	-	-
F6P transketolase (12)	0.007	0.007	0.974	0	1.000	0
SBP aldolase (13)	0	0	0	-	-	-
SBPase (14)	0	0	0	-	-	-
S7P transketolase (15)	0.006	0.007	0.974	0	1.000	0
Ru5P epimerase (16)	0.013	0.013	0.974	0	1.000	0
R5P isomerase (17)	0.006	0.007	0.973	0	1.000	0
Ru5P kinase (18)	0.019	0.020	0.974	0	1.000	0
PGA kinase [Backward] (7)	0	0	0	-	-	-
TP isomerase [Backward] (9)	0	0	0	-	-	-
FBP aldolase [Backward] (10)	0.004	0.145	0.025	0	1.000	0
Starch metabolism						
starch synthase (22)	2.949e-04	2.949e-04	1.000	0	0	1.000
starch synthase (23)	2.975e-04	3.068e-04	0.970	0	1.000	0
starch synthase (24)	2.610e-06	1.177e-05	0.222	0	1.000	0
amylase (26)	2.610e-06	2.107e-05	0.124	0	1.000	0
disproportionating enzyme (28)	0	9.165e-06	0	0	1.000	0
disproportionating enzyme (29)	0	9.298e-06	0	0	1.000	0

Table S4. A comparison of the maximum alternative optimal flux values of G and M cells for the reactions depicted in Figure 1. Results derived from the Flux Variability Analysis applied to the alternative optima space of RegrEx: $V_{max}G$, $V_{max}M$, the maximum flux value in the alternative optima space (as calculated by $RegrEx_{FVA}$) in G and M cells, respectively, and their difference are included. The p-values shown in this table correspond to a Mann-Whitney test comparing the distributions of flux values of G and M cells (V_G and V_M, respectively).

**Idx. in Fig.1**	**Reaction Name (Idx in AraCOREred)**	**$V_{max}G$**	**$V_{max}M$**	**$V_{max}G$- $V_{max}M$**	**Ho: $V_G > V_M$**	**SubSystem**
PSII	photosystem II (1)	0.125	0.125	0	0.841	light reactions
Cb6f	cytochrom b6f complex (2)	0.250	0.250	0	0.841	light reactions
PSI	photosystem I (3)	0.500	0.500	0	0.841	light reactions
ATPase	ATPase (5)	0.107	0.107	0	0.841	light reactions
1	PEP carboxylase (54)	0.466	0.054	0.412	1.000	gluconeogenesis
2	Cytosolic NADP-MDH (115)	0.168	0.105	0.063	1.000	pyruvate metabolism
3	Dicarboxylate transporter (339)	0.255	0.054	0.201	1.000	transport
4	Plastidial NADP-Malic Enzyme (113)	0.254	0.053	0.201	1.000	pyruvate metabolism
5	CO2 diffusion [Forward] (374)	1.000 e-06	0.012	-1.158e-02	0	transport
5	CO2 diffusion [Backward] (374)	0.234	0.033	0.201	1.000	transport
6	Import CO2 (413)	0.021	0.021	4.950e-05	0.006	import
7	Carbonic anhydrase (152)	0.466	0.054	0.412	1.000	carbon fixation
8	Glu synthase (FeS-Fd) (179)	0.250	0.026	0.224	1.000	glutamate synthesis
9	ferredoxin-NADP reductase (4)	0.235	0.250	-1.529e-02	0	light reactions
10	Export O2 (420)	0.020	0.020	1.310e-04	0.576	export
11	TP isomerase [Forward] (9)	0.874	0.924	-5.007e-02	0	Calvin-Benson cycle, glycolysis
12	TP/Pi translocator [Forward] (328)	0.888	1.000	-1.117e-01	0	transport
13	Di-/ri-carboxylate carrier [Forward] (346)	1.000	1.000	0	1.000	transport
13	Di-/ri-carboxylate carrier [Forward] (347)	1.000	1.000	0	1.000	transport
13	Di-/ri-carboxylate carrier [Forward] (348)	1.000	1.000	0	1.000	transport
14	Di-/ri-carboxylate carrier [Backward] (343)	1.000	1.000	0	1.000	transport
14	Di-/ri-carboxylate carrier [Backward] (344)	1.000	1.000	0	1.000	transport
14	Di-/ri-carboxylate carrier [Backward] (345)	1.000	1.000	0	1.000	transport
15	Mitochondrlial NAD-MDH [Backward] (80)	0.323	0.323	-4.698e-04	1.000	tricarboxylic acid cycle, glyoxylate cycle
15	Mitochondrial NADP-MDH (117)	0.769	0.746	0.023	1.000	pyruvate metabolism
16	FBP aldolase [Forward] (35)	0.034	0.218	-1.839e-01	0	sucrose synthesis, gluconeogenesis, glycolysis
17	FBPase (36)	0.090	0.006	0.083	1.000	sucrose synthesis, gluconeogenesis, glycolysis
17	PPi-dep. Phosphofructokinase [Backward] (136)	1.000 e-06	0.695	-6.950e-01	0	pyrophosphate recycling
18	G6P isomerase [Forward] (39)	0.034	0.218	-1.839e-01	0	sucrose synthesis, sucrose degradation, gluconeogenesis
19	Phosphoglucomutase [Forward] (40)	0.057	0.003	0.054	1.000	sucrose synthesis, sucrose degradation
20	TP/Pi translocator [Forward] (327)	1.000 e-06	1.000e-06	0	1.000	transport
20	TP/Pi translocator [Backward] (327)	0.386	0.600	-2.147e-01	0	transport

Table S5. A comparison of the maximum alternative optimal flux values of G and M cells for the reactions in the CBC and starch metabolism. Results derived from the Flux Variability Analysis applied to the alternative optima space of RegrEx: $V_{max}G$, $V_{max}M$, the maximum flux value in the alternative optima space (as calculated by $RegrEx_{FVA}$) in G and M cells, respectively, and their difference are included. The p-values shown in this table correspond to a Mann-Whitney test comparing the distributions of flux values of G and M cells (V_G and V_M, respectively).

Reaction [direction](Idx in GEM)	$V_{max}G$	$V_{max}M$	$V_{max}G$- $V_{max}M$	Ho: $V_G > V_M$
CBC				
RuBisCO Carboxylation (6)	0.021	0.023	-1.784e-03	0
RuBisCO Oxygenation(85)	2.514e-04	2.661e-04	-1.467e-05	-
PGA kinase [Forward] (7)	0.500	0.299	0.201	1.000
GAP dehydrogenase (8)	0.911	0.963	-5.196e-02	0
TP isomerase [Forward] (9)	0.874	0.924	-5.007e-02	0
FBP aldolase [Forward] (10)	0.005	1.000e-06	0.005	1.000
FBPase (11)	0.005	0.005	1.373e-04	1.96E-01
F6P transketolase (12)	0.007	0.008	-5.059e-04	0
SBP aldolase (13)	0.005	0.006	-8.402e-04	0
SBPase (14)	0.005	0.006	-8.402e-04	0
S7P transketolase (15)	0.007	0.008	-5.058e-04	0
Ru5P epimerase (16)	0.014	0.015	-1.012e-03	0
R5P isomerase (17)	0.007	0.007	-5.058e-04	0
Ru5P kinase (18)	0.021	0.023	-1.784e-03	0
PGA kinase [Backward] (7)	1.000e-06	1.000e-06	0	-
TP isomerase [Backward] (9)	1.000e-06	1.000e-06	0	0
FBP aldolase [Backward] (10)	0.020	0.204	-1.838e-01	0
Starch metabolism				
starch synthase (22)	0.020	0.204	-1.838e-01	1.000
starch synthase (23)	0.002	0.002	6.434e-04	0
starch synthase (24)	0.005	0.006	-7.696e-04	0
amylase (26)	0.004	0.003	0.001	0
disproportionating enzyme (28)	0.004	0.003	0.001	0
disproportionating enzyme (29)	0.004	0.003	0.001	0
disproportionating enzyme (30)	0.004	0.003	0.001	1.76E-05
(starch) phosphorylase (32)	0.002	0.002	1.543e-04	1.000

Table S6. A comparison of the predicted metabolic state of G and M cells after imposing additional experimental constraints. This table shows the analogous results, presented inTable S1, when additional constraints are taken in consideration. Concretely, the carboxylation to oxygenation ratio of RubisCO is constrained to stay within 1.5 and 4. Additionally, the flux through the reactions in the CBC: the *sedoheptulose 1,7-bisphosphate aldolase* and the *sedoheptulose-1,7-bisphosphatase* is constrained to carry a positive flux (Details in Materials & Methods). A horizontal bar indicates a failure of the test due to distributions consisting of a fixed value. Reaction names and index numbers in accord with AraCOREred.

Idx in Fig 1	Reaction Name (Idx in AraCOREred)	Mean Flux G	Mean Flux M	Mean ratio (G/M)	Ho: $V_G = V_M$	Ho: $V_M > V_G$	Ho: $V_G > V_M$	SubSystem
PSII	photosystem II (1)	0.125	0.125	1.000	-	-	-	light reactions
Cb6f	cytochrom b6f complex (2)	0.250	0.250	1.000	-	-	-	light reactions
PSI	photosystem I (3)	0.500	0.500	1.000	-	-	-	light reactions
ATPase	ATPase (5)	0.107	0.107	1.000	-	-	-	light reactions
1	PEP carboxylase (54)	0.420	0.011	37.190	0	0	1.000	gluconeogenesis
2	Mal dehydrogenase (115)	0.161	0.086	1.866	0	0	1.000	pyruvate metabolism
3	Dicarboxylate transporter (339)	0.223	0.011	19.971	0	0	1.000	transport
4	Mal dehydrogenase (113)	0.221	0.009	24.697	0	0	1.000	pyruvate metabolism
5	CO2 diffusion [Forward] (374)	0	0.013	0	0	1.000	0	transport
5	CO2 diffusion [Backward] (374)	0.199	7.085e-08	2.812e+06	0	0	1.000	transport
6	Import CO2 (413)	0.021	0.021	1.000	0	1.000	0	import
7	HCO3 dehydratase (152)	0.420	0.011	37.190	0	0	1.000	carbon fixation
8	Glu synthase (FeS-Fd) (179)	0.120	1.670e-04	719.020	0	0	1.000	glutamate synthesis
9	ferredoxin-NADP reductase (4)	0.130	0.250	0.520	0	1.000	0	light reactions
10	Export O2 (420)	0.020	0.020	1.000	0	1.000	0	export
11	TP isomerase [Forward] (9)	0.854	0.825	1.035	0	0	1.000	Calvin-Benson cycle, glycolysis
12	TP/Pi translocator [Forward] (328)	0.856	1.000	0.856	0	1.000	0	transport
13	Di-/ri-carboxylate carrier [Forward] (346)	0.362	0.335	1.079	0	0	1.000	transport
13	Di-/ri-carboxylate carrier [Backward] (346)	0.355	0.335	1.060	0	0	1.000	transport
13	Di-/ri-carboxylate carrier [Forward] (347)	0.362	0.338	1.069	0	0	1.000	transport
13	Di-/ri-carboxylate carrier [Backward] (347)	0.332	0.309	1.076	0	0	1.000	transport
13	Di-/ri-carboxylate carrier [Forward] (348)	0.327	0.310	1.056	1.455e-05	7.273e-06	1.000	transport
13	Di-/ri-carboxylate carrier [Backward] (348)	0.332	0.305	1.089	0	0	1.000	transport
14	Di-/ri-carboxylate carrier [Forward] (343)	0.249	0.208	1.199	0	0	1.000	transport
14	Di-/ri-carboxylate carrier [Backward] (343)	0.753	0.720	1.046	0	0	1.000	transport
14	Di-/ri-carboxylate carrier [Forward] (344)	0.022	0.194	0.113	0	1.000	0	transport
14	Di-/ri-carboxylate carrier [Backward] (344)	0.058	0	Inf	0	0	1.000	transport
14	Di-/ri-carboxylate carrier [Forward] (345)	0	0.693	0	0	1.000	0	transport
14	Di-/ri-carboxylate carrier [Backward] (345)	0.022	0.194	0.113	0	1.000	0	transport
15	Mal dehydrogenase [Backward] (80)	0.036	7.590e-04	47.157	0	0	1.000	tricarboxylic acid cycle, glyoxylate cycle
15	Mal dehydrogenase (117)	0	0	0	-	-	-	pyruvate metabolism
16	FBP aldolase [Forward] (35)	0.394	0.405	0.973	0	1.000	0	sucrose synthesis, gluconeogenesis, glycolysis
17	FBPase (36)	0.125	0.125	1.000	-	-	-	sucrose synthesis, gluconeogenesis, glycolysis
17	PPi-dep. Phosphofructokinase [Backward] (136)	0.250	0.250	1.000	-	-	-	pyrophosphate recycling
18	G6P isomerase [Forward] (39)	0.500	0.500	1.000	-	-	-	sucrose synthesis, sucrose degradation, gluconeogenesis
19	Phosphoglucomutase [Forward] (40)	0.107	0.107	1.000	-	-	-	sucrose synthesis, sucrose degradation
20	TP/Pi translocator [Forward] (327)	0.420	0.011	37.190	0	0	1.000	transport
20	TP/Pi translocator [Backward] (327)	0.161	0.086	1.866	0	0	1.000	transport

Table S7. Activity of the CBC and starch metabolism in G and M cells after imposing additional experimental constraints. This table shows the analogous results to that of Table S3, when additional constraints are taken in consideration. Concretely, the carboxylation to oxygenation ratio of RubisCO is constrained to stay within 1.5 and 4. Additionally, the flux through the reactions in the CBC: the *sedoheptulose 1,7-bisphosphate aldolase* and the *sedoheptulose-1,7-bisphosphatase* is constrained to carry a positive flux (Details in Materials & Methods). A horizontal bar indicates a failure of the test due to distributions consisting of a fixed value. Reaction names and index numbers in accord with AraCOREred.

Reaction [direction](Idx in GEM)	Mean Flux G	Mean Flux M	Mean ratio (G/M)	Ho: $V_G = V_M$	Ho: $V_M > V_G$	Ho: $V_G > V_M$
CBC						
RuBisCO Carboxylation (6)	0.022	0.022	1.000	0	1.000	0
RuBisCO Oxygenation(85)	0.005	0.005	1.000	0	1.000	0
PGA kinase [Forward] (7)	0.467	0.255	1.832	0	0	1.000
GAP dehydrogenase (8)	0.864	0.959	0.901	0	1.000	0
TP isomerase [Forward] (9)	0.854	0.825	1.035	0	0	1.000
FBP aldolase [Forward] (10)	5.544e-04	0	Inf	0	0	1.000
FBPase (11)	0.001	0.001	1.000	-	-	-
F6P transketolase (12)	0.009	0.009	1.000	0	1.000	0
SBP aldolase (13)	0.001	0.001	1.000	-	-	-
SBPase (14)	0.001	0.001	1.000	-	-	-
S7P transketolase (15)	0.009	0.009	1.000	0	1.000	0
Ru5P epimerase (16)	0.018	0.018	1.000	0	1.000	0
R5P isomerase (17)	0.009	0.009	1.000	0	1.000	0
Ru5P kinase (18)	0.027	0.027	1.000	0	1.000	0
PGA kinase [Backward] (7)	0	0	0	-	-	-
TP isomerase [Backward] (9)	0	0	0	-	-	-
FBP aldolase [Backward] (10)	0.004	0.176	0.021	0	1.000	0
Starch metabolism						
starch synthase (22)	2.949e-04	2.949e-04	1.000	0	0	1.000
starch synthase (23)	2.975e-04	3.065e-04	0.971	0	1.000	0
starch synthase (24)	2.632e-06	1.203e-05	0.219	0	1.000	0
amylase (26)	2.632e-06	2.106e-05	0.125	0	1.000	0
disproportionating enzyme (28)	2.239e-08	9.423e-06	0.002	0	1.000	0
disproportionating enzyme (29)	0	9.031e-06	0	0	1.000	0

Table S8. Predicted Flux-Sums of selected metabolites in the AraCOREred model after imposing additional experimental constraints. This table presents the analogous results of Table S2 when additional constraints are taken in consideration. Concretely, the carboxylation to oxygenation ratio of RubisCO is constrained to stay within 1.5 and 4. Additionally, the flux through the reactions in the CBC: the *sedoheptulose 1,7-bisphosphate aldolase* and the *sedoheptulose-1,7-bisphosphatase* is constrained to carry a positive flux (Details in Materials & Methods).

Metabolite (compartment)	Mean FluxSum G	Mean FluxSum M	Ratio (G/M)	Ho: $V_G = V_M$	Ho: $V_M > V_G$	Ho: $V_G > V_M$
Total Mal	5.615	4.722	1.189	0	0	1.000
Mal(c)	2.678	2.359	1.135	0	0	1.000
Mal(m)	2.355	2.186	1.077	0	0	1.000
Mal(p)	0.006	0.006	1.001	0	1.000	0
Mal(h)	2.155	1.443	1.493	0	0	1.000
Total Suc	0.070	5.574e-05	1.258e+03	0	0	1.000
Futile Cycle Suc	0.070	1.203e-05	5.822e+03	0	1.000	0
Total OAA	7.570	6.777	1.117	0	0	1.000
OAA(c)	4.191	3.677	1.140	0	0	1.000
OAA(m)	2.156	2.017	1.069	0	0	1.000
OAA(p)	1.326	1.494	0.888	0	1.000	0
OAA(h)	1.713	1.425	1.202	0	0	1.000
Total Pyr	3.510	2.980	1.178	0	0	1.000
Pyr(c)	1.413	1.303	1.084	0	0	1.000
Pyr(h)	0.442	0.057	7.725	0	0	1.000
Pyr(m)	1.656	1.639	1.010	0	0	1.000
Pyr(p)	1.413	1.264	1.117	0	0	1.000
Total PEP	2.926	2.542	1.151	0	0	1.000
PEP(c)	1.937	1.679	1.154	0	0	1.000
PEP(h)	1.538	1.672	0.920	0	1.000	0
G3P(h)	3.348	2.539	1.318	0	0	1.000
G3P(c)	1.693	0.845	2.003	0	0	1.000
Total ATP	1.802	1.417	1.271	0	0	1.000
ATP(h)	0.968	0.968	1.000	0	0	1.000
ATP(c)	6.691	6.226	1.075	0	0	1.000
ATP(m)	3.445	3.344	1.030	0	0	1.000
Total NADP	1.740	1.442	1.207	0	0	1.000
NADP(h)	1.506	1.440	1.046	0	0	1.000
NADP(c)	6.691	6.226	1.075	0	0	1.000
NADP(m)	3.445	3.344	1.030	0	0	1.000
Total NADPH	1.740	1.442	1.207	0	0	1.000
NADPH(h)	1.506	1.440	1.046	0	0	1.000
NADPH(c)	2.759	1.619	1.704	0	0	1.000
NADPH(m)	2.116	1.580	1.339	0	0	1.000
Total CO_2	0.839	0.048	17.473	0	0	1.000
CO_2(h)	0.007	0.007	1.000	0	1.000	0
CO_2(c)	5.615	4.722	1.189	0	0	1.000
CO_2(m)	2.678	2.359	1.135	0	0	1.000

Table S9. Redistribution of the percentage of ^{13}C label enrichment in primary metabolites. M and G cells were fed with ^{13}-$NaHCO_3$ and harvested after 30 min and 60 min in the light. Values in bold and underline type are significantly different between M and G cells according to Students *t*-test ($P < 0.05$) in the same time point. Data presented are mean ± SE (n = 3).

		% of 13C enrichment							
**Metabolite**	**m/z**	**M30**	**SE**	**M60**	**SE**	**GC30**	**SE**	**GC60**	**SE**
**Gly**	**102**	0.010	0.004	0.005	0.001	0.015	0.003	0.006	0.002
**Ser**	**306**	0.016	0.014	0.009	0.005	0.007	0.004	**<u>0.036</u>**	**<u>0.004</u>**
**Ser**	**204**	0.101	0.052	0.060	0.020	0.111	0.036	0.097	0.007
**Homoserine**	**128**	0.449	0.272	0.380	0.028	0.282	0.124	0.332	0.237
**Glycolate**	**205**	0.008	0.008	0.007	0.007	0.015	0.008	0.017	0.008
**Val**	**218**	0.240	0.180	0.095	0.091	0.051	0.027	0.162	0.035
**Ala**	**188**	0.416	0.198	0.361	0.141	1.476	1.260	0.459	0.355
**Thr**	**219**	0.458	0.238	0.342	0.132	0.795	0.330	0.634	0.067
**Thr**	**291**	0.927	0.438	0.748	0.260	1.489	0.447	**<u>1.361</u>**	**<u>0.118</u>**
**Pro**	**142**	0.197	0.100	0.164	0.021	0.321	0.151	**<u>0.367</u>**	**<u>0.012</u>**
**Asp**	**218**	0.018	0.018	0.012	0.012	**<u>1.638</u>**	**<u>0.947</u>**	0.786	0.622
**Asp**	**232**	0.045	0.004	0.037	0.002	**<u>0.078</u>**	**<u>0.005</u>**	0.038	0.001
**Leu**	**158**	0.034	0.000	0.033	0.000	0.031	0.002	0.034	0.002
**Ile**	**218**	0.038	0.038	0.091	0.091	0.068	0.049	0.203	0.101
**Glu**	**156**	0.010	0.001	0.014	0.004	0.020	0.009	0.022	0.007
**Glycerate**	**292**	1.676	0.636	1.922	0.653	**<u>3.521</u>**	**<u>0.069</u>**	3.609	0.746
**Lactate**	**117**	0.111	0.075	0.421	0.033	0.344	0.172	0.268	0.163
**Glycerol**	**293**	0.072	0.041	0.081	0.043	0.075	0.055	0.114	0.018
**Succ**	**172**	0.176	0.080	0.283	0.100	**<u>0.752</u>**	**<u>0.154</u>**	**<u>0.506</u>**	**<u>0.044</u>**
**Succ**	**247**	0.219	0.071	0.266	0.102	**<u>0.954</u>**	**<u>0.071</u>**	**<u>0.607</u>**	**<u>0.039</u>**
**Mal**	**233**	0.040	0.001	0.053	0.010	**<u>0.100</u>**	**<u>0.001</u>**	0.041	0.002
**Fum**	**245**	0.098	0.026	0.084	0.014	0.094	0.033	0.128	0.019
**GABA**	**174**	0.015	0.003	0.024	0.008	nd	nd	nd	nd
**Erythritol**	**217**	0.425	0.130	0.438	0.149	2.212	1.808	1.207	0.871
**Sucrose**	**437**	0.000015	0.000007	0.000018	0.000009	0.000104	0.000068	0.000036	0.000010

Table S10. Total ^{13}C-enrichment in primary metabolites. Experimental and statistical analysis as described in the Table S9.

	Total 13C enrichment								
**Metabolite**	**m/z**	**M30**	**SE**	**M60**	**SE**	**GC30**	**SE**	**GC60**	**SE**
**Gly**	**102**	3257	1665	4837	624	1083	496	4738	997
**Ser**	**306**	71.7	22.1	194.9	40.0	135.6	90.9	**303.9**	**23.4**
**Ser**	**204**	1642	976	1786	579	921	332	836	103
**Homoserine**	**128**	1524	976	628	44	1331	526	2202	1073
**Glycolate**	**205**	658	658	646	646	1212	609	1370	595
**Val**	**218**	30.3	7.2	20.9	13.7	**7.3**	**4.7**	13.2	0.9
**Ala**	**188**	694	513	715	335	479	228	69	36
**Thr**	**219**	276	105	270	75	136	38	128	13
**Thr**	**291**	735	253	740	201	400	104	377	49
**Pro**	**142**	251	92	186	24	133	50	**52**	**15**
**Asp**	**218**	12.0	12.0	12.5	12.5	14.8	1.7	8.9	4.7
**Asp**	**232**	1533	76	1303	37	**2352**	**133**	1154	6
**Leu**	**158**	499	23	514	29	**386**	**31**	437	33
**Ile**	**218**	1.84	1.84	8.88	8.88	2.72	1.36	17.59	7.21
**Glu**	**156**	6938	109	6594	2023	**2249**	**566**	2423	313
**Glycerate**	**292**	92.6	37.0	78.0	15.7	39.9	0.3	47.9	8.9
**Lactate**	**117**	356	251	148	13	349	130	500	230
**Glycerol**	**293**	3363	1273	2824	992	5972	2954	1522	283
**Succ**	**172**	5380	3759	1361	336	779	246	905	165
**Succ**	**247**	303	88	226	64	**87**	**12**	134	22
**Mal**	**233**	1341	31	1790	330	**3242**	**41**	1289	53
**Fum**	**245**	713	241	651	113	548	179	529	19
**GABA**	**174**	14330	2650	9640	3556	nd	nd	nd	nd
**Erythritol**	**217**	206	53	186	43	133	64	**60**	**15**
**Sucrose**	**437**	477177	82661	607521	270298	427991	174100	486175	190880

Table S11. ^{13}C-enrichment in primary metabolites. Experimental and statistical analysis as described in Table S9.

	13C enrichment								
**Metabolite**	***m/z***	**M30**	**SE**	**M60**	**SE**	**GC30**	**SE**	**GC60**	**SE**
**Gly 2TMS**	**102**	4.7	0.2	5.1	0.1	3.6	0.7	5.0	0.2
**Ser**	**306**	0.8	0.5	1.1	0.2	1.0	0.6	**3.3**	**0.1**
**Ser**	**204**	8.9	0.6	9.2	0.4	8.9	0.5	8.9	0.2
**Homoserine**	**128**	16.1	0.3	15.4	0.1	15.9	0.5	**16.5**	**0.0**
**Glycolate**	**205**	2.3	2.3	2.1	2.1	4.3	2.1	4.8	2.1
**Val**	**218**	2.5	1.3	1.3	1.1	0.6	0.3	1.5	0.2
**Ala**	**188**	9.4	1.5	12.1	4.2	12.8	1.4	5.1	2.9
**Thr**	**219**	8.9	0.2	8.5	0.1	9.1	0.4	**8.9**	**0.0**
**Thr**	**291**	21.6	0.5	21.2	0.3	22.5	0.1	22.4	0.5
**Pro**	**142**	5.6	0.1	5.4	0.0	**5.3**	**0.0**	4.3	0.6
**Asp**	**218**	0.5	0.47	0.4	0.39	**4.4**	**1.4**	2.3	1.3
**Asp**	**232**	8.3	0.6	7.0	0.3	**13.5**	**0.8**	6.6	0.1
**Leu**	**158**	4.1	0.1	4.1	0.1	**3.5**	**0.3**	3.9	0.2
**Ile**	**218**	0.3	0.3	0.9	0.9	0.4	0.2	1.9	0.9
**Glu**	**156**	6.3	1.6	7.8	0.4	5.1	2.4	**5.0**	**1.0**
**Glycerate**	**292**	10.5	0.3	11.4	0.6	**11.9**	**0.1**	12.6	0.1
**Lactate**	**117**	5.3	2.6	7.9	0.6	8.8	0.2	8.1	0.2
**Uracil**	**241**	5.2	0.2	5.6	0.5	**7.1**	**0.0**	6.4	0.2
**Uracil**	**255**	9.1	0.1	9.3	0.1	**10.8**	**0.4**	**10.8**	**0.1**
**Adipic acid**	**111**	1.9	0.1	1.9	0.1	1.8	0.0	2.1	0.1
**Adipic acid**	**141**	6.0	0.6	5.9	0.2	6.0	0.3	6.5	0.3
**Threonate**	**292**	10.0	0.2	10.1	0.4	6.5	3.7	nd	nd
**Salicylic acid**	**267**	8.8	1.2	9.3	1.1	nd	nd	nd	nd
**Glycerol**	**293**	12.1	0.6	12.0	0.5	12.4	0.1	12.8	0.2
**OXA**	**175**	nd	nd	nd	nd	14.5	0.3	nd	nd
**Succ**	**172**	22.6	6.1	17.8	0.5	22.5	1.7	**21.0**	**1.1**
**Succ**	**247**	7.3	0.5	6.9	0.3	**9.0**	**0.3**	**9.0**	**1.0**
**Mal**	**233**	7.3	0.0	9.7	1.8	**18.0**	**0.1**	7.3	0.3
**Citramalate**	**247**	0.3	0.28	0.1	0.097	0.8	0.4	**1.3**	**0.8**
**Fum**	**245**	7.6	0.4	7.2	0.0	6.7	1.6	8.2	0.5
**GABA**	**174**	6.6	0.1	6.4	0.1	nd	nd	nd	nd
**Erythritol**	**217**	8.6	0.2	8.3	0.2	9.3	1.0	6.6	1.8
**myo inositol**	**191**	4.9	4.9	4.8	4.8	7.2	4.1	11.6	2.1
**Sucrose**	**437**	2.5	0.8	2.5	0.1	**4.7**	**0.3**	**3.7**	**0.4**
**Trehalose**	**169**	3.9	2.2	5.6	2.8	5.1	2.7	5.0	2.5
**Maltose**	**361**	7.6	3.8	8.7	2.2	nd	nd	8.1	0.3
**Isomaltose**	**361**	nd	nd	12.6	0.6	nd	nd	nd	nd

Table S12. Content of the metabolites analyzed in this study. The content (ng) of each metabolite was obtained using the equation of a linear regression of the peak area obtained from different concentrations of standard compounds.

	Concentration (ng)							
**Metabolite**	**M30**	**SE**	**M60**	**SE**	**GC30**	**SE**	**GC60**	**SE**
**Gly**	675.1	319.4	949.3	103.7	266.1	84.7	959.5	238.7
**Ser**	171.7	94.6	189.2	55.6	100.6	32.8	93.5	9.3
**Homoserine**	92.3	57.6	40.9	2.8	81.7	30.6	133.6	65.2
**Glycolate**	291.5	7.3	290.9	6.1	283.0	4.0	284.3	2.8
**Val**	17.1	4.7	29.4	8.4	9.6	2.1	9.3	0.7
**Ala**	71.7	54.4	58.7	32.7	42.1	22.1	23.4	8.4
**Thr**	34.5	12.5	34.8	9.3	17.8	4.7	16.8	1.8
**Pro**	44.2	15.9	34.3	4.3	24.8	9.4	**<u>11.7</u>**	**<u>2.0</u>**
**Asp**	184.4	3.9	187.1	3.9	**<u>174.1</u>**	**<u>0.9</u>**	**<u>175.0</u>**	**<u>1.5</u>**
**Leu**	120.6	2.3	124.9	3.7	111.3	0.8	112.8	1.5
**Ile**	17.2	6.2	22.5	6.3	9.9	2.8	10.0	0.7
**Glu**	673.4	207.5	697.1	263.6	224.6	92.0	324.1	146.4
**Glycerate**	8.9	3.7	7.0	1.7	3.4	0.0	3.8	0.7
**Lactate**	46.4	29.2	18.7	0.9	40.1	15.5	60.7	27.5
**Salicylic acid**	10.7	7.6	5.3	2.2	0.5	0.1	0.5	0.1
**Glycerol**	289.4	118.7	243.6	93.2	482.3	239.2	118.2	20.1
**Succ**	192.6	93.8	77.4	20.6	33.3	8.4	42.5	5.7
**Mal**	182.9	4.3	184.3	1.1	179.6	1.9	177.7	0.1
**Fum**	94.9	33.1	90.4	15.7	81.0	22.4	**<u>65.8</u>**	**<u>7.0</u>**
**GABA**	489.1	97.1	343.7	120.0	**<u>135.4</u>**	**<u>78.2</u>**	**<u>35.2</u>**	**<u>20.3</u>**
**Erythritol**	24.1	6.6	22.7	5.5	15.8	8.0	11.5	4.7
**Sucrose**	246540	86181	239692	101307	97619	43785	124067	39544

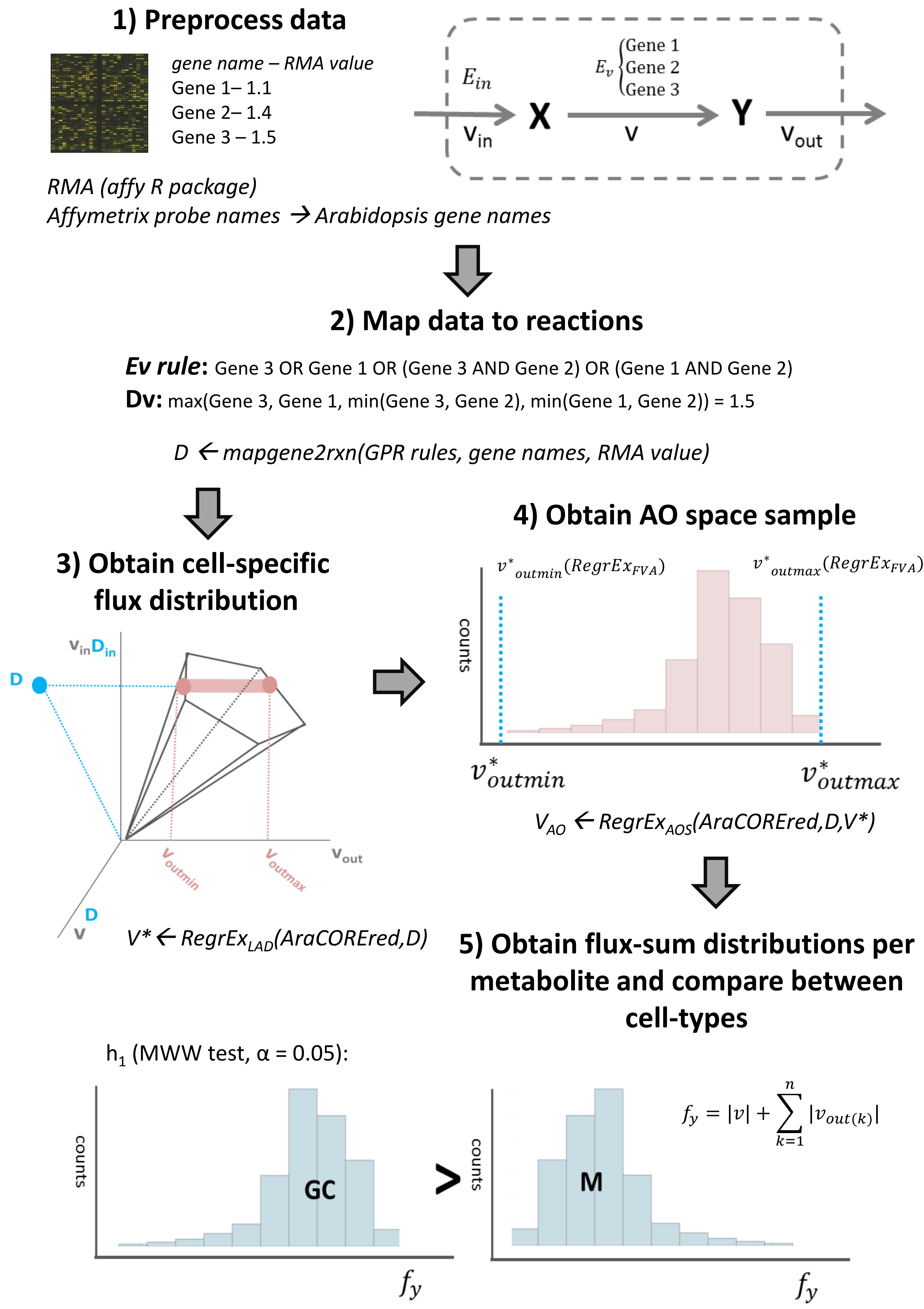

1) Preprocess data
gene name – RMA value
Gene 1– 1.1
Gene 2– 1.4
Gene 3 – 1.5
RMA (affy R package)
Affymetrix probe names → Arabidopsis gene names
E_{in}
E_v Gene 1 Gene 2 Gene 3
v_{in}
X
v
Y
v_{out}
2) Map data to reactions
Ev rule: Gene 3 OR Gene 1 OR (Gene 3 AND Gene 2) OR (Gene 1 AND Gene 2)
Dv: max(Gene 3, Gene 1, min(Gene 3, Gene 2), min(Gene 1, Gene 2)) = 1.5
D ← mapgene2rxn(GPR rules, gene names, RMA value)
3) Obtain cell-specific flux distribution
$v_{in}D_{in}$
D
v_{outmin}
v_{outmax}
v_{out}
D
v
$V^* \leftarrow RegrEx_{LAD}(AraCOREred,D)$
4) Obtain AO space sample
$v^*_{outmin}(RegrEx_{FVA})$
$v^*_{outmax}(RegrEx_{FVA})$
counts
v^*_{outmin}
v^*_{outmax}
$V_{AO} \leftarrow RegrEx_{AOS}(AraCOREred,D,V^*)$
5) Obtain flux-sum distributions per metabolite and compare between cell-types
h_1 (MWW test, α = 0.05):
counts
GC
>
counts
M
$f_y = |v| + \sum_{k=1}^{n} |v_{out(k)}|$
f_y
f_y

Figure S1. Schematic depiction of the workflow followed to obtain metabolic predictions specific to G and M cells. This schematic depiction is based on the toy metabolic model displayed in the top right: X first enters the system through the reaction, v_{in}, which is dependent on the transporter E_{in}. X is then transformed to Y through v, which is dependent on E_v (coded by gene1-3). Finally Y diffuses spontaneously to the exterior (v_{out}), and hence no genes are associated to this reaction. (1) In a first step, expression data is preprocessed, which includes data RMA normalization and mapping of array probe names to (Arabidopsis) gene names. (2) Gene expression values are then mapped to reactions in the metabolic model following the gene-protein-reaction rules contained in the model, which generates the vector D of mapped expression values. (3) In a third step, a cell-specific flux distribution that is closest to the mapped expression data integrated in the metabolic model is obtained through $RegrEx_{LAD}$. However, the optimal solution (*i.e.,*flux distribution) is not unique, and an alternative optima space (AO) exists. In this case, this is because D contains data to only two of the three reactions (v_{in} and v) since v_{out} has not associated gene-protein-reaction rule. Thus, v_{out} can vary in an orthogonal direction to the plane, where D lies, without affecting the optimal value of the $RegrEx_{LAD}$ objective function (see Appendix S1). (4) To account for this issue, the AO space is sampled through $RegrEx_{AOS}$ and a sampled distribution of optimum flux values for v_{out} is obtained. Additionally, the function RegrExFVA calculates the minimum and maximum values in the alternative optima space, as a means to validate the coverage of the random sample. (5) Finally, flux-sums are calculated for each metabolite across the previously obtained AO sample (here only depicted for the case of Y), thus obtaining another distribution of flux-sums. This process is applied to the two cell types, G and M cells, and the resulting AO flux and flux-sum distributions are compared through a Mann-Whitney-Wilcoxon (MWW) test. In this example, the alternative hypothesis (H1) states that the flux-sum distribution corresponding to G cells is greater than that of M cells, while the null hypothesis states that both distributions are indistinguishable. Here, the null hypothesis is rejected at a significant level of $\alpha = 0.05$.

Appendix S1. A brief description of the RegrEx Alternative Optima Sampling method used in this study

RegrEx [1] finds the optimal flux distribution within the flux cone, $v_{opt} \in C = \{v: Sv = 0, v_{min} \leq v \leq v_{max}\}$, that minimizes the second norm of the difference vector $\epsilon = d - |v|$, where d represents the data vector used (ϵ is defined only over the set of reactions with associated data, R_D in OP_1). This is achieved through the mixed integer quadratic program displayed in OP_1. The introduction of the vector of binary variables, x, is required to cope with the absolute values (*i.e.*, $|v|$) in ϵ in a manner that guarantees the computational tractability of the optimization problem. To this end, reversible reactions in v are split into the forward and backward direction (v_{for} and v_{back}, respectively) and forced to take non-negative values and be mutually exclusive (*i.e.* for each reversible reaction i, $v_{for(i)} \geq 0 \; XOR \; v_{back(i)} \geq 0$), as ensured by constraints 6-9 in OP_1.

$$
\begin{aligned}
& v_{opt} = \underset{\substack{\epsilon=[\epsilon_{irr};\epsilon_{for};\epsilon_{back}]\in\mathbb{R} \\ v=[v_{irr};v_{for};v_{back}]\in\mathbb{R}_0^+, \\ x\in\{0,1\}^n}}{\arg\min} \tfrac{1}{2}\|\epsilon\|_2^2 + \lambda \| v \|_1 \\
& s.t. \\
& 1.\ Sv = 0 \\
& \left.\begin{aligned}
& 2.\ v_{irr_i} + \epsilon_{irr} = d_{irr} \\
& 3.\ v_{for_i} + \epsilon_{for} - x_i d_{rev} = d_{rev} \\
& 4.\ v_{back_i} + \epsilon_{back} - x_i d_{rev} = 0
\end{aligned}\right\}, \quad i \in R_D \qquad (OP_1). \\
& 5.\ v_{irr\min} \leq v_{irr} \leq v_{irr\max} \\
& 6.\ v_{for} + x v_{for\min} \geq v_{for\min} \\
& 7.\ v_{back} - x v_{back\min} \geq 0 \\
& 8.\ v_{for} + x v_{for\max} \leq v_{for\max} \\
& 9.\ v_{back} - x v_{back\max} \leq 0
\end{aligned}
$$

In addition to the second norm of ϵ, a penalty term weighted by parameter λ is added to the objective function. This penalty term corresponds to the first norm of the flux vector, v, which controls the sparsity in v_{opt} as a means to eliminate reactions that are not important (as determined by the used expression data) to a given context.

In this study, we have slightly modified the objective function in OP_1. Concretely, we minimized the first norm of ϵ instead of its second norm. This change converts the previous mixed integer quadratic program to the mixed integer linear program displayed in OP_2.

$$
\begin{aligned}
& v_{opt} = \arg\min_{\substack{\epsilon^{+}=[\epsilon^{+}_{irr};\epsilon^{+}_{for};\epsilon^{+}_{back}],\\ \epsilon^{-}=[\epsilon^{-}_{irr};\epsilon^{-}_{for};\epsilon^{-}_{back}],\\ v=[v_{irr};v_{for};v_{back}]\in\mathbb{R}^{+}_{0},\\ x\in\{0,1\}^{n}}} \mathrm{w}^{T}(\epsilon^{+}+\epsilon^{-}) + \lambda \| v \|_1 \\
& s.t. \\
& 1.\ S_{ext} v = 0 \\
& \left.\begin{aligned}
& 2.\ v_{irr_i} + (\epsilon^{+}_{irr} - \epsilon^{-}_{irr}) = d_{irr} \\
& 3.\ v_{for_i} + (\epsilon^{+}_{for} - \epsilon^{-}_{for}) + x d_{rev} = d_{rev} \\
& 4.\ v_{back_i} + (\epsilon^{+}_{back} - \epsilon^{-}_{back}) - x d_{rev} = 0
\end{aligned}\right\}, \quad i \in R_D \\
& 5.\ v_{irr\min} \le v_{irr} \le v_{irr\max} \qquad\qquad (\mathrm{OP}_2). \\
& 6.\ v_{for} + x v_{for\min} \ge v_{for\min} \\
& 7.\ v_{back} - x v_{back\min} \ge 0 \\
& 8.\ v_{for} + x v_{for\max} \le v_{for\max} \\
& 9.\ v_{back} - x v_{back\max} \le 0 \\
& 10.\ r_{lb} v_O - v_C \le 0 \\
& 11.\ r_{ub} v_O - v_C \ge 0
\end{aligned}
$$

The first norm of ϵ involves absolute values (*i.e.*, $\|\epsilon\|_1 = \sum_i |\epsilon_i|$), which cannot be directly treated in conventional numerical solvers. A way to computationally deal with this consist of splitting ϵ into two non-negative terms (*i.e.* $\epsilon = \epsilon^{+} - \epsilon^{-}$). Hence, $\|\epsilon\|_2^2$ is replaced by $\epsilon^{+} + \epsilon^{-}$ in the objective function of OP_2.In addition, a vector w, weighting the contribution of each element in ϵ, was introduced in the objective function Concretely, each element $w_i = \sigma_i^{-1}$ was defined as the inverse value of the standard deviation σ_i of the gene expression among the three replicates available for each cell type that was associated to reaction *i*. The last two constraints (10 and 11) impose the constraint on the carboxylation to oxygenation ratio discussed in the main text, *i.e.* $r_{lb} \le \frac{v_C}{v_O} \le r_{ub}$.

Finally, the development of the modified RegrEx version (called $\mathrm{RegrEx}_{\mathrm{LAD}}$, for least absolute deviations) presented in OP_2 was motivated to facilitate the computational tractability of the alternative optima space associated to the data integration problem (next section).

Evaluation of the alternative optima space

When integrating expression data into a genome-scale model, an alternative optima space, V_{AO}, of optimal flux distributions, v_{opt}, may exist. Concretely, when using OP_2 for the data integration, the elements of V_{AO} share the same objective function value and satisfy all imposed constraints, that is, $V_{AO} = \{v_{opt}: \|\epsilon\|_1 + \lambda\|v_{opt}\|_1 = Z_{opt},\ Sv_{opt} = 0\ , v_{min} \le v_{opt} \le v_{max}\}$. The main cause of this alternative optimal space of solutions can be attributed to the existence of reactions in a genome-scale model that do not contain associated gene expression data. This subsequently implies that there are reactions whose flux can vary without affecting the value of Z_{opt}.

We used a recently developed method [2], to analyze the alternative optima space associated to the data integration in AraCOREred. $\mathrm{RegrEx}_{\mathrm{AOS}}$ (RegrEx Alternative Optima Sampling) was

designed to generate a uniform sample of the alternative optima space associated to $\mathrm{RegrEx_{LAD}}$. To this end, it first generates a random flux vector, v_{rand}, and then searches for the closest flux distribution, $v \in V_{AO}$, to v_{rand} that belongs to the alternative optima space of the previous $\mathrm{RegrEx_{LAD}}$ optimization. This is accomplished through the mixed integer linear program depicted in $\mathrm{OP_3}$ (this process is repeated *n* times to obtain the uniform sample).

$$
\begin{aligned}
&\min_{\substack{\epsilon^{+}=[\epsilon^{+}_{irr};\epsilon^{+}_{for};\epsilon^{+}_{back}],\\ \epsilon^{-}=[\epsilon^{-}_{irr};\epsilon^{-}_{for};\epsilon^{-}_{back}],\\ \delta^{+}=[\delta^{+}_{irr};\delta^{+}_{for};\delta^{+}_{back}],\\ \delta^{-}=[\delta^{-}_{irr};\delta^{-}_{for};\delta^{-}_{back}],\\ v=[v_{irr};v_{for};v_{back}]\in\mathbb{R}^{+}_{0},\\ x\in\{0,1\}^{n}}} \quad \| \delta^{+} + \delta^{-} \|_1 \\
&s.t. \\
&1\text{–}11 \quad (\mathrm{OP_2}) \\
&12.\ w^{T}(\epsilon^{+} + \epsilon^{-}) = w^{T}(\epsilon^{+}_{opt} + \epsilon^{-}_{opt}) \\
&13.\ \| v \|_1 = \| v_{opt} \|_1 \\
&14.\ v_{irr} - (\delta^{+}_{irr} - \delta^{-}_{irr}) = v_{rand(irr)} \\
&15.\ v_{for} - (\delta^{+}_{for} - \delta^{-}_{for}) - x v_{rand(rev)} = 0 \\
&16.\ -v_{back} + \delta_{back} + x v_{rand(rev)} = v_{rand(rev)}
\end{aligned}
\qquad (\mathrm{OP_3}).
$$

$\mathrm{OP_3}$ inherits constraints 1-11 from $\mathrm{OP_2}$ and introduces two sets of new constraints. One set is formed by constraints 12 and 13, and guarantees that the sampled flux distribution shares the same objective value than the previously found v_{opt} in $\mathrm{OP_2}$. The other set consists of constraints 14-16 introducing the auxiliary variables $\delta = \delta^{+} - \delta^{-} = v_{rand} - v^{*}_{opt}$, which measure the distance of an alternative optimum flux distribution to the randomly generated v_{rand}. In addition, δ is partitioned into the set of irreversible and the forward and reverse directions of reversible reactions, which simplifies the computation of the first norm of δ in the objective function.

In addition to generating a sample of alternative optima flux distributions with $\mathrm{RegrEx_{AOS}}$, we also developed a complementary approach to evaluate the extreme flux values within the alternative optima space. This approach, akin to the Flux Variability Analysis [3] procedure, minimizes and maximizes the flux v_j through each reaction *j* in the genome-scale metabolic model such that the flux distribution remains in the alternative optima space, that is, $v \in V_{AO}$. Computing the extreme flux values within the alternative optima space serves to assess the quality of the sampling performed by $\mathrm{RegrEx_{AOS}}$. Concretely, it allows evaluating whether the sample covers the whole range of flux values within the alternative optima space—*i.e.*, it is uniform. This evaluation is importance, since a sample covering the whole allowable range renders not only statistically significant, but also quantitatively accurate results. This is fundamental when comparing alternative optimal distributions of flux values between different metabolic contexts. Our complementary approach, which we term $\mathrm{RegrEx_{FVA}}$ (RegrEx Flux Variability Analysis), iterates the mixed integer linear program in $\mathrm{OP_4}$ through each reaction *j* in the metabolic model (one time minimizing v_j , the other maximizing it).

Constraints 1-11 are again inherited from OP_1, and constraints 12 and 13, which ensure $v \in V_{AO}$, are identical to the corresponding ones in OP_3.

$$\begin{aligned} & \min_{\substack{\epsilon^+ = [\epsilon^+_{irr}; \epsilon^+_{for}; \epsilon^+_{back}], \\ \epsilon^- = [\epsilon^-_{irr}; \epsilon^-_{for}; \epsilon^-_{back}], \\ v = [v_{irr}; v_{for}; v_{back}] \in \mathbb{R}^+_0, \\ x \in \{0,1\}^n}} / \max \quad v_j \\ & s.t. \\ & 1-11 \quad (OP_2) \\ & 12.\ w^T (\epsilon^+ + \epsilon^-) = w^T (\epsilon^+_{opt} + \epsilon^-_{opt}) \\ & 13.\ \| v \|_1 = \| v_{opt} \|_1 \end{aligned} \qquad (OP_4).$$

References:

1. Robaina Estévez, S. & Nikoloski, Z. Context-Specific Metabolic Model Extraction Based on Regularized Least Squares Optimization. *PLoS One* **10,** e0131875 (2015).

2. Robaina Estévez, S. & Nikoloski, Z. On the effects of alternative optima in context-specific metabolic model predictions. Plos Comp.Biol. *In Press.* (2017).

3. Mahadevan, R. & Schilling, C. H. The effects of alternate optimal solutions in constraint-based genome-scale metabolic models. *Metab. Eng.* **5,** 264–276 (2003).
